# Lessons From a Systematic Literature Search on Diagnostic DNA Methylation Biomarkers for Colorectal Cancer: How to Increase Research Value and Decrease Research Waste?

**DOI:** 10.14309/ctg.0000000000000499

**Published:** 2022-05-18

**Authors:** Zheng Feng, Cary J. G. Oberije, Alouisa J. P. van de Wetering, Alexander Koch, Kim. A. D. Wouters, Nathalie Vaes, Ad A. M. Masclee, Beatriz Carvalho, Gerrit A. Meijer, Maurice P. Zeegers, James G. Herman, Veerle Melotte, Manon van Engeland, Kim M. Smits

**Affiliations:** 1 Department of Pathology, GROW – School for Oncology and Reproduction, Maastricht University Medical Center, Maastricht, the Netherlands;; 2 The D-Lab, Department of Precision Medicine, GROW—School for Oncology and Reproduction, Maastricht University Medical Centre, Maastricht, the Netherlands;; 3 Division of Gastroenterology and Hepatology, Department of Internal Medicine, Maastricht University Medical Center, Maastricht, the Netherlands;; 4 Division of Gastroenterology-Hepatology, Department of Internal Medicine, NUTRIM School of Nutrition and Translational Research in Metabolism, Maastricht University Medical Centre, Maastricht, the Netherlands;; 5 Department of Pathology, Netherlands Cancer Institute, Amsterdam, the Netherlands;; 6 Department of Complex Genetics, NUTRIM School of Nutrition and Translational Research in Metabolism, Maastricht University Medical Center, Maastricht, the Netherlands;; 7 Department of Complex Genetics, CAPHRI – Care and Public Health Research Institute, Maastricht University Medical Center, Maastricht, the Netherlands;; 8 Division of Hematology/Oncology, University of Pittsburgh Cancer Institute, Pittsburgh, Pennsylvania, USA;; 9 Department of Clinical Genetics, Erasmus University Medical Center, University of Rotterdam, Rotterdam, the Netherlands;; 10 Division of Medical Oncology, Department of Internal Medicine, GROW – School for Oncology and Reproduction, Maastricht University Medical Center, Maastricht, the Netherlands.

## Abstract

To improve colorectal cancer (CRC) survival and lower incidence rates, colonoscopy and/or fecal immunochemical test screening are widely implemented. Although candidate DNA methylation biomarkers have been published to improve or complement the fecal immunochemical test, clinical translation is limited. We describe technical and methodological problems encountered after a systematic literature search and provide recommendations to increase (clinical) value and decrease research waste in biomarker research. In addition, we present current evidence for diagnostic CRC DNA methylation biomarkers.

**METHODS:**

A systematic literature search identified 331 diagnostic DNA methylation marker studies published before November 2020 in PubMed, EMBASE, Cochrane Library, and Google Scholar. For 136 bodily fluid studies, extended data extraction was performed. STARD criteria and level of evidence were registered to assess reporting quality and strength for clinical translation.

**RESULTS:**

Our systematic literature search revealed multiple issues that hamper the development of DNA methylation biomarkers for CRC diagnosis, including methodological and technical heterogeneity and lack of validation or clinical translation. For example, clinical translation and independent validation were limited, with 100 of 434 markers (23%) studied in bodily fluids, 3 of 434 markers (0.7%) translated into clinical tests, and independent validation for 92 of 411 tissue markers (22%) and 59 of 100 bodily fluids markers (59%).

**DISCUSSION:**

This systematic literature search revealed that major requirements to develop clinically relevant diagnostic CRC DNA methylation markers are often lacking. To avoid the resulting research waste, clinical needs, intended biomarker use, and independent validation should be better considered before study design. In addition, improved reporting quality would facilitate meta-analysis, thereby increasing the level of evidence and enabling clinical translation.

## INTRODUCTION

Colorectal cancer (CRC) is the third most common cancer worldwide and a major contributor to cancer-related mortality (1). In 2018, the incidence was 1.8 million and over 881.000 people died from CRC (2). By 2030, this is expected to increase to over 2.2 million new cases and 1.1 million deaths (3), illustrating the heavy patient and societal burden (4). Early detection and treatment of CRC and its high-risk precursor lesions leads to reduced mortality, morbidity, and healthcare costs (5). Therefore, CRC screening programs have been widely implemented (6).

Currently, colonoscopy is the gold standard method to diagnose CRC, allowing obtaining biopsies and resecting qualifying (pre)cancerous lesions directly. However, it is invasive with a high patient burden, mainly caused by the required bowel preparation and postcolonoscopy abdominal symptoms (7). Occasionally, severe complications occur, for example, bleeding (0.26%) and perforation (0.05%) (8).

Colonoscopy numbers increase rapidly with screening, inherently yielding an increase in surveillance demand after the removal of (pre)cancerous lesions (9). This already consumes a large part of the total colonoscopy capacity, depending on the total available capacity in a specific country (10,11). To lower the burden for patients and clinicians and avoid complications, preselection of patients for colonoscopy should be optimized.

The most common preselection test is the fecal immunochemical test (FIT), measuring stool hemoglobin levels (6). However, because FIT sensitivities are suboptimal, especially for advanced adenomas (12,13) and early-stage cancers (14,15), and false-positive rates can be high (18), there is an ongoing search for biomarkers to complement FIT (19,20). Many candidate biomarkers have been reported, amongst which DNA methylation markers (21–24) in tissue, blood, urine, or stool (25–27). However, only 3 CRC detection tests measuring DNA methylation markers are currently commercially available: Cologuard (*NDRG4* and *BMP3* methylation combined with occult hemoglobin and *KRAS* mutations)*,* Epi proColon/ColoVantage (licensed to Quest Diagnostics and ARUP Laboratories), and Epi proColon (both SEPTIN9 methylation) (28). This contrast between the number of published candidate biomarkers and the number of available clinical tests, illustrates the gap between discovery and clinical implementation.

To improve clinical translation of diagnostic CRC DNA methylation markers, it is important to understand the clinical needs, the status of previous research, and to know which markers have a diagnostic accuracy that might improve currently used tests. Our systematic literature search was performed to provide this evidence. In addition, we aimed to assess specific issues that still hinder the clinical development of potential DNA methylation biomarkers and define recommendations to overcome these issues.

## METHODS

### Eligibility criteria, search strategy, and selection

**Figure 1.**
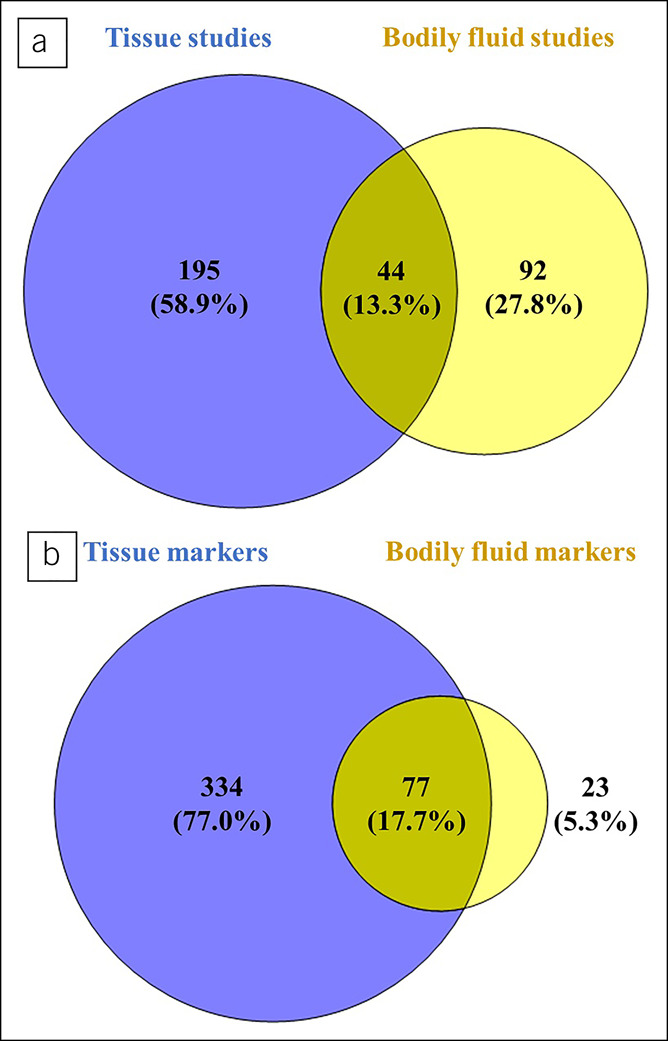
Venn diagram of studies and markers. (**a**) *Article overview*: Among 331 articles, there were 195 articles focusing on tissue, 92 articles on bodily fluid samples, and 44 studies using both tissue and bodily fluid samples. **(b)** *Marker overview*: A total of 331 articles generated 434 biomarkers. 334 markers were studied in tissue, 23 markers in bodily fluid samples, and 77 markers in both tissue and bodily fluid samples.

A systematic review was performed to identify studies on diagnostic CRC DNA methylation markers, applying the Preferred Reporting Items for Systematic Review and Meta-Analysis of Diagnostic Test Accuracy Studies guidelines for the literature search (29). Originally, English language articles were considered if patients or cancer tissues were compared with healthy controls or noncancerous tissues; reviews, editorials, and conference abstracts were excluded. Inclusion was not restricted to specific study designs, patient characteristics, tumor subtype, disease stage, or included patient number. Studies on hereditary cancer syndromes or studies evaluating genome-wide methylation instead of gene-specific alterations were excluded because these were outside the scope of this review. PubMed, EMBASE, Cochrane Library, and Google Scholar were searched up to November 1, 2020, with no additional limits defined (see Supplementary Table 1, Supplementary Digital Content 1, http://links.lww.com/CTG/A814). Reference lists of included articles were searched for additional relevant publications. Finally, 331 articles were included. Among these, 195 focused on tissue, 92 on bodily fluid samples, and 44 on both tissue and bodily fluids (Figure 1).

### Data extraction

Five researchers (ZF, KS, AvdW, CO, and KW) performed data extraction, using a standardized data registration form. Each article was assessed by 2 researchers independently. If discrepancies arose, a third researcher was involved to reach consensus. Restricted data extraction (author, year of publication, methylation markers, and specimen type) was performed for all 331 studies. Full data extraction (study design, patient characteristics, disease severity, tumor location, DNA isolation and DNA methylation method, primer/probe sequences, and diagnostic measures) was performed for 136 articles on bodily fluid markers because only noninvasive diagnostic markers are considered as clinically relevant (see Supplementary Figure 1 and Supplementary Table 2, Supplementary Digital Content 1, http://links.lww.com/CTG/A814). Not all included studies seemed to be specifically designed to develop a diagnostic CRC DNA methylation biomarker, and some of these early-phase studies contribute useful information to the scientific knowledge base on DNA methylation in CRC. However, most of these studies made claims on the diagnostic potential of their studied biomarker and reported a biomarker potential in the title (99/136; 73%) or the discussion (124/136; 91%).

### Reporting quality and level of evidence

STARD 2015 criteria were adjusted for biomarker research and used to assess reporting quality (30) (see Supplementary Table 3, Supplementary Digital Content 1, http://links.lww.com/CTG/A814). One point was assigned for reported items, 0.5 points for incomplete reported items, and zero points if items were not reported. The maximum score was 22 points. STARD scores were also used to analyze reporting quality of specific categories (i.e., population selection, assay method, outcome assessment, and other variable assessment) (see Supplementary Tables 3 and 4, Supplementary Digital Content 1, http://links.lww.com/CTG/A814). If a study scored ≥0.5 points per item for STARD items 5–9, reporting of population selection was considered as sufficient; a score of <0.5 indicated increased risk of bias. Other reporting categories were evaluated similarly, using STARD items 10a, 12a, and 13a for the assay method and STARD items 14, 21a, and 24 for the outcome assessment. The other variable assessment was based on STARD item 20. If this item was not reported, variable assessment was insufficient. With a full or partial score, this was assessed as sufficient. To grade the level of evidence (LoE), we used a ranking scheme adapted for biomarkers (30,31) and the Oxford Centre for Evidence-Based Medicine 2011 Levels of Evidence (32).

## RESULTS

### Diagnostic CRC biomarkers throughout the years

The first report on DNA methylation markers in tissue was published in 1985. Since then, numbers have been increasing (see Supplementary Figure 2, Supplementary Digital Content 1, http://links.lww.com/CTG/A814); we identified 331 studies on 434 DNA methylation markers (see Supplementary Figures 2a and 2b). Of these, 334 of the 434 markers (77.0%) were investigated in tissue, 77 of the 434 markers (17.7%) in tissue and bodily fluids, and 23 of the 434 markers (5.3%) in bodily fluids. The number of published tissue markers showed a peak in 2016 and declined thereafter. This might reflect a shift of interest to bodily fluids as these peak after 2016, or a shift to other biomarker types that were not assessed in this review (Figure 1).

The first report on DNA methylation markers in bodily fluids (TMEFF2 [TPEF]) appeared in 2003 (see Supplementary Figure 2b, Supplementary Digital Content 1, http://links.lww.com/CTG/A814). Since then, 136 studies (41.1%) reported diagnostic DNA methylation markers in bodily fluids while 195 studies (58.9%) focused solely on tissue (see Supplementary Figures 2a and 2b, Supplementary Digital Content 1, http://links.lww.com/CTG/A814). This illustrates that, despite the growing interest in noninvasive markers, many studies still solely focus on tissue. Part of these studies might not have been designed to assess a biomarker potential but rather to assess the role of DNA methylation in CRC pathogenesis and contribute additional information to the scientific knowledge base of DNA methylation in CRC. The majority, however, did make (strong) claims on a biomarker potential for their DNA methylation marker.

**Figure 2.**
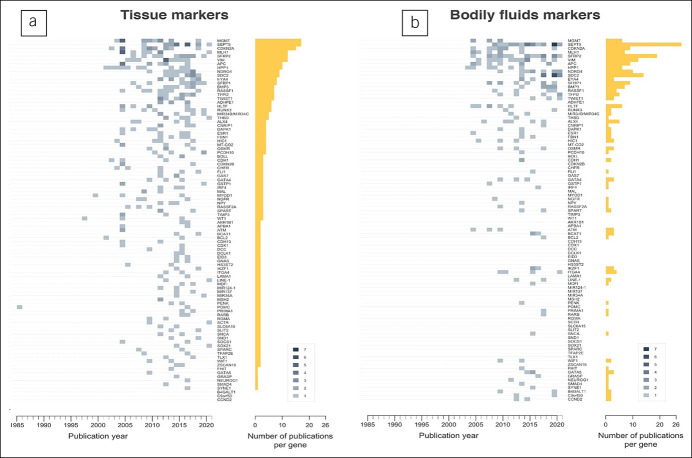
Number of yearly publications for the tissue-based **(a)** and body fluid-based **(b)** colorectal cancer DNA methylation biomarkers studied more than once; darker colors reflect a higher number of publications for the specified biomarker in a particular year. The gliding box indicates the publication count by color. The length of bars represents the total number of publications of the specified biomarker from 1985 to 2020.

Next to lack of translation from tissue to bodily fluids, independent validation (i.e., evaluating biomarkers in other studies or study populations) rarely occurred. In tissue, 92 of 411 markers (22.4%) were studied at least twice; *CDKN2A (p16)* and *MGMT* were most frequently studied (Figure 2a). In bodily fluids, 59 of 100 markers (59.0%) were studied more than once with *SEPTIN9* as the most frequently studied marker in 34 studies and *SFRP2*, *SDC2*, *VIM*, and *NDRG4* in 20, 14, 11, and 10 studies, respectively (Figure 2b). By contrast, 12 other markers were only validated twice indicating that even if independent validation was performed, this was performed in a limited number of validation sets (33). Internal validation was often performed by splitting sample sets, but, especially in small populations, bootstrapping might be more suitable (34).

Independent validation was unrelated to initial results. For *MGMT* stool methylation, for example, initial results did not seem promising, with 14.1% sensitivity and 79.6% specificity. Nevertheless, *MGMT* was studied in 5 subsequent studies showing sensitivities between 5.7% and 90.0%. These large ranges could probably not be fully explained by differences in tumor or patient characteristics, such as cancer stage or tumor location. Technical aspects such as sample preparation or collection, assay design, and technological resolution differences also influence these results. By contrast, markers showing more promising initial results, although in small sample sets, for example, *SNCA* (studied in 89 cases and 30 controls; sensitivity 70%, specificity 100%), have not been independently validated yet.

**Figure 3.**
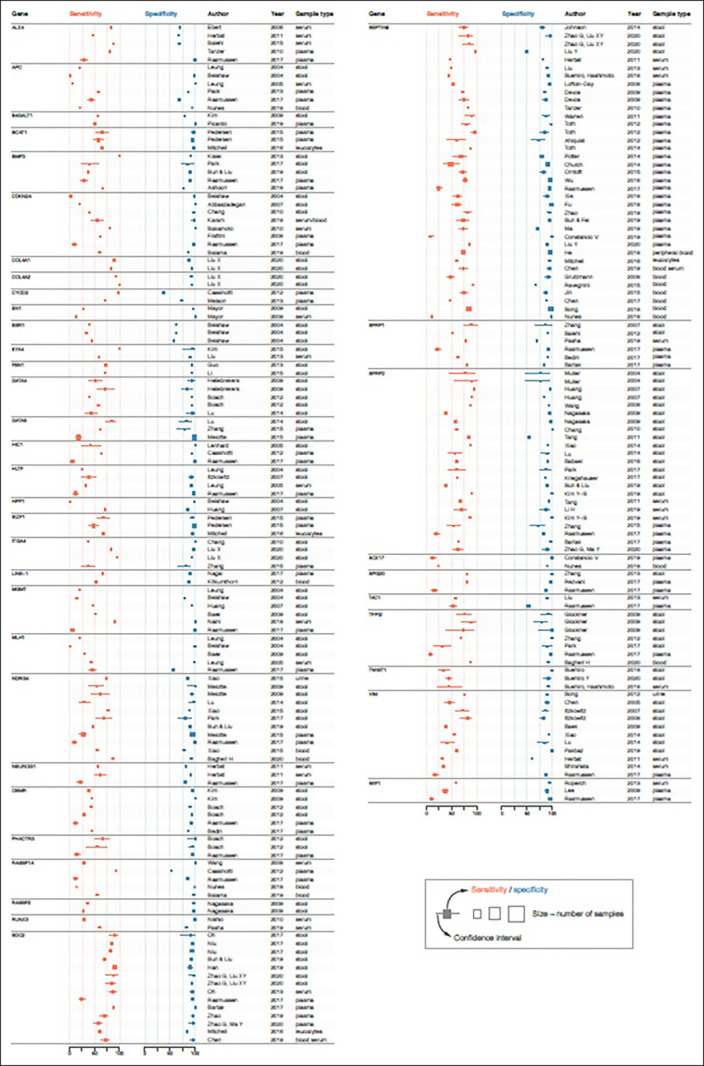
Forest plots of reported DNA methylation markers in colorectal cancer studies.

Independent validation is crucial in biomarker research (35) to verify initial findings that are often too optimistic because of multiple testing in a limited data set (36). *SFRP2* methylation in stool, for example, was first reported as promising, with 90%–94% sensitivity and 77%–95% specificity, but subsequent studies yielded sensitivities between 20% and 90.5% and specificities between 54% and 100% (Figure 3). Although optimism correction approaches have been described (37), external validation studies, with a similar design and similar technical assays as used in the initial study, are necessary to assess the expected future range of sensitivities and specificities. To ultimately draw conclusions on diagnostic biomarker performance and sufficiently increase LoE, large prospective clinical trials or meta-analyses are necessary (30–32). However, owing to variations in study design, patient selection, sample preparation, test characteristics, and outcome assessment, meta-analysis is often unfeasible. In addition, the quality and usefulness of meta-analyses completely depend on the input data quality, emphasizing the importance of individual study and technical assay quality.

Many studies in this review used different biomarker assays; often it was unclear whether these measured the most relevant DNA methylation signal. In addition, suboptimal technical assays, poorly specific primers, and suboptimal DNA methylation locations were used. Over recent years, an increasing number of studies attempt to identify DNA methylation biomarkers using publicly available data such as The Cancer Genome Atlas. Although these studies are not included in this systematic review, their potential to identify novel diagnostic biomarkers should be acknowledged (28). On the other hand, it might be difficult to externally validate potential diagnostic signatures consisting of many CpGs or to implement these in clinical practice. In addition, the cost-effectiveness of these signatures could also be questioned.

### Overview of CRC biomarkers in bodily fluids

Of 136 included bodily fluids studies, 51 (37.5%) focused on stool; 77 (56.6%) on blood (plasma, serum, whole blood, or cells derived from blood); 1 on urine (<1%); 6 on stool and blood (4.4%); and 1 on blood, urine, and stool (<1%) (see Supplementary Table 5, Supplementary Digital Content 1, http://links.lww.com/CTG/A814).

Forest plots were constructed for 41 bodily fluid markers studied in more than 2 studies or populations (Figure 3). Next to reasons mentioned earlier (e.g., flaws in study design, lack of validation, and initial optimism of results), variations in sensitivity and specificity could also be caused by methodological differences between studies (e.g., patient or sample selection and preparation, technical assays, statistical approaches, and varying sample sizes). This is, for example, shown not only for *VIM* methylation, with sensitivities between <20% and 80% and specificities <90%, but also for other markers, such as *APC*, *MLH1*, and *RASSF1A* (Figure 3).

### Comparison with currently implemented clinical tests

From all biomarkers in this review, probably only a small, selected group holds sufficient potential to warrant further validation. However, it remains challenging to identify this group. An important issue is the additional value as compared with the current noninvasive test for early CRC detection (FIT). Because FIT is already widely accepted and implemented, a biomarker, alone or in a panel, should add value or be more cost-effective than FIT, otherwise the chances of clinical translation will be small. For CRC, FIT has an estimated sensitivity between 68% and 87% and a specificity of 96%–98% (17,38). For advanced adenomas, sensitivity is substantially lower, 23%–40% (10,39), leaving room for improvement. From markers studied more than once, 17 of the 41 markers (41.5%; *APC*, *B4GALT1*, *BCAT1*, *EN1*, *ESR1*, *HIC1*, *HLTF*, *LINE-1*, *MLH1*, *NEUROG1*, *OSMR*, *RASSF2*, *RUNX3*, *SOX17*, *TAC1, TWIST1*, and *WIF-1*) did not report a sensitivity as good as FIT nor did they evaluate marker performance in addition to FIT. Although specificity ranges seem to be more in line with FIT, performance is less for some markers (*ALX4*, *BMP3*, *COL4A1*, *COL4A2*, *ESR1*, *SFRP1*, *TAC1*, and *VIM)*. Even for established DNA methylation markers that are used in clinical tests (*NDRG4* and *BMP3* in Cologuard) or have been launched as alternative for FIT (*SEPTIN9* in Epi proColon/ColoVantage and Epi proColon (28)), some published sensitivities or specificities are lower than those of FIT (Figure 3). The fact that these established DNA methylation markers have shown an incremental diagnostic value, however, indicates that sensitivity and/or specificity alone should not be used to draw final conclusions on the diagnostic biomarker value. Instead, this conclusion might only be drawn after evaluating candidates, alone or multimarker panels, in predictive models also containing FIT. In addition, it needs to be emphasized that the choice of a suitable population is crucial in biomarker research. For CRC early detection biomarkers, only studies conducted in screening asymptomatic participants can actually evaluate the diagnostic performance of the biomarker. Symptomatic participants are less useful for this purpose because they probably represent a different spectrum of the disease and more advanced stages, as compared with asymptomatic participants. Biomarkers identified in symptomatic patient groups might be useful to distinguish (late-stage) cancer cases from controls but might be less relevant to identify CRC cases in a screening setting.

### Quality of reporting

**Figure 4.**
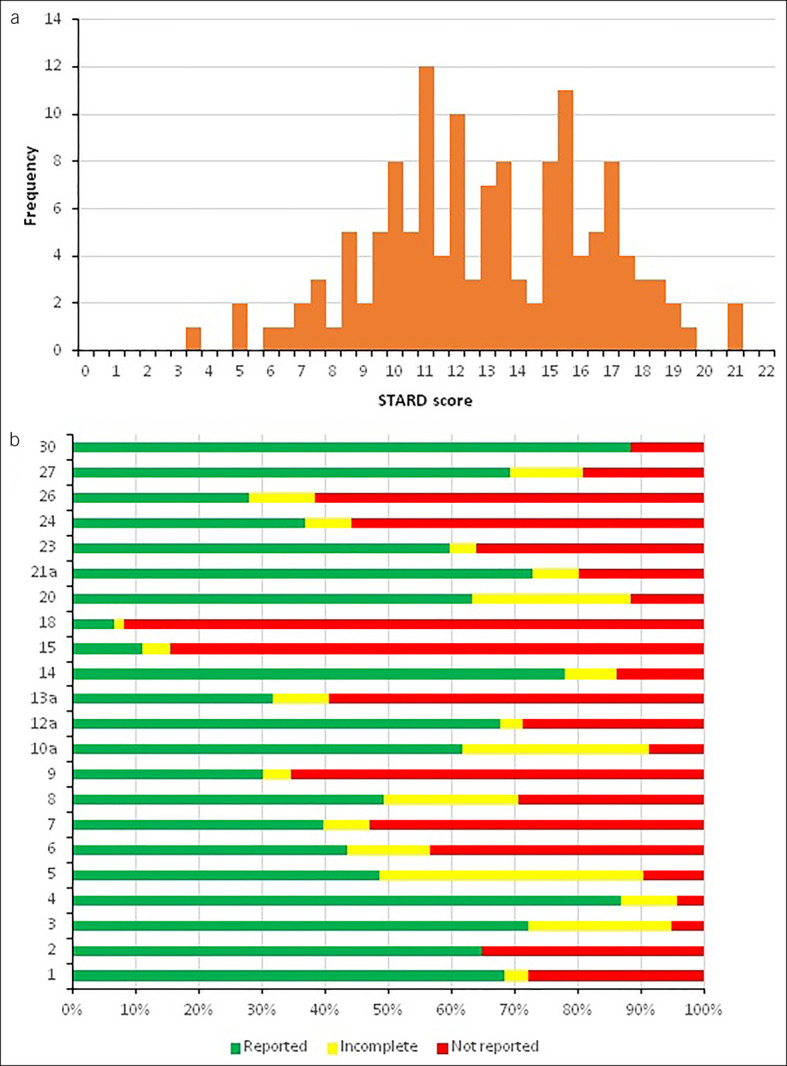
Quality assessment (STARD) of 136 articles reporting bodily fluid-based DNA methylation biomarkers for CRC. **(a)** Histogram depicting the STARD score distribution for bodily fluid-based biomarker studies (mean STARD score = 13.0, SD = 3.5). **(b)** Stacked bar chart showing the percentage of completeness of each STARD item. CRC, colorectal cancer.

STARD scores for the 136 included studies ranged from 3.5 to 21 points of 22 points maximum (median 13.5) (Figure 4a) (30). Items 3 (intended biomarker use), 4 (study objectives), 5 (data collection), and 10a (assay method) were completely or partially described in >90% of the studies, whereas items 15 (handling of missing biomarker data) and 18 (sample size/power) were reported in less than 20% (Figure 4b). This shows that although STARD criteria are widely accepted, actual adherence to STARD is poor.

Several items were not scored using the STARD criteria because these items would not have given additional information. These items include item 11 (reference rationale), because there is no alternative for colonoscopy, and item 25 (adverse events), because direct adverse events for biomarker testing are absent or negligible. In addition, other items might need more emphasis because these are essential for data interpretation or experimental replication. These include details on all laboratory methods and cutoff determination, not only the DNA methylation test (index test) and detailed descriptions of sample collection, processing, and storage. Therefore, we believe that redefining current STARD criteria to better serve biomarker studies would improve uniform, high-quality reporting of these studies and facilitate meta-analyses.

Poor reporting is not synonymous to poor research or a useless biomarker, but it severely hampers validation studies and meta-analyses. Because independent external validation is limited, the performance of large clinical trials is costly and complicated, and the inclusion of sufficient cases is challenging; systematic reviews and meta-analyses are necessary to increase the LoE for potential biomarkers. However, for meta-analysis, high individual study quality is crucial.

### Problems in study design and methodology

Problems previously described to hinder efficient clinical translation (e.g., methodological heterogeneity and poor reporting quality) (35,40–43) were also observed in this systematic review (see Supplementary Table 4, Supplementary Digital Content 1, http://links.lww.com/CTG/A814) next to other problems such as technical heterogeneity.

Large sample size variations were observed across studies; the smallest counted 10 subjects and the largest 801 (Supplementary Table 5, Supplementary Digital Content 1, http://links.lww.com/CTG/A814); sample size justification was lacking in 124 of the 136 studies (91.2%). Although small sample sizes are not synonymous for low quality, the risk of false-positive findings is significantly larger (44), and multiple small studies should be combined to draw appropriate conclusions on the biomarker value. However, owing to large interstudy heterogeneity, this is often impossible, resulting in a constantly low LoE for those markers without prospects to clinical translation (see Supplementary Table 4, Supplementary Digital Content 1, http://links.lww.com/CTG/A814). Moreover, even if studies could theoretically be combined, many likely suffer from (selection) bias; combining them would therefore not yield a representative sample of the target population. In addition, most studies include subjects referred for colonoscopy; biomarker sensitivity will probably decrease when applied to the general screening population. Specificity, however, might actually be higher as compared with the study setting (45).

Next to selection bias and inappropriate or small populations, also publication bias could lead to an overestimation of the diagnostic performance of a biomarker and hinder reproducibility or meta-research on biomarkers. The fact that significant or favorable results are more likely to be published is well recognized. Despite efforts to prevent publication bias, such as registries or journals specifically also accepting null findings, the reality is that publication bias still represents a ubiquitous problem in many scientific fields (46,47). Publication bias is expected to have occurred in the field of diagnostic DNA methylation biomarkers as well, leading to selective reporting of findings on these biomarkers.

Next to methodological heterogeneity, technical heterogeneity has similar consequences. Within 136 included studies, analytic methods could be categorized in 19 different approaches to measure methylation. Quantitative methylation-specific PCR was most widely used (55 studies; 40.4%), followed by methylation-specific PCR (48 studies; 35.3%). Especially if not optimized for the specific research question, diverse technical assays can yield different results (48,49). Often, different experimental settings (40,50,51) were used, including different DNA input and cutoffs for quantitative results. Overall, 14 methods of cutoff determination for quantitative data were reported but often without information on the rationale and without determining minimal criteria for sensitivity and specificity a priori. When subsequently comparing several biomarkers or biomarker panels, with each other, often changes in sensitivities were reported accompanied by a decline in specificity. However, to be able to fairly compare biomarkers, it can be questioned whether it should not be preferred to adjust the cutoff to achieve comparable specificities or work with a predetermined specificity for all biomarkers based on the envisioned clinical application (52,53).

This heterogeneity can hinder interstudy comparisons and consequently hamper improvement of LoE and clinical translation. Nevertheless, when carefully selected and optimized, different technical assays can yield comparable results (54). Different techniques could therefore be used for biomarker validation purposes, but proper and detailed experimental reporting is essential.

## DISCUSSION

### The way forward: How to bring diagnostic CRC DNA methylation markers to the clinic?

**Figure 5.**
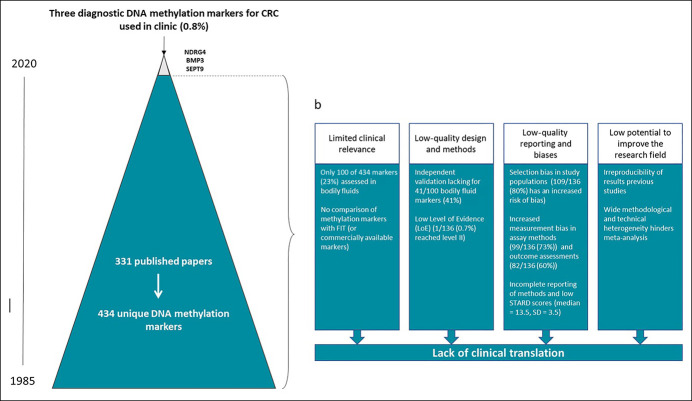
Gap between colorectal cancer methylation marker research and clinical implementation. (**a**) The clinical translation rate of DNA methylation markers for early diagnosis of CRC (0.8%) was calculated by comparing the number of commercially available biomarkers with the total number of published biomarkers (from 1985 to 2020). **(b)** Four stages of waste in the development and reporting of DNA methylation marker research in CRC relevant to clinicians and patients (adapted from avoidable waste in the production and reporting of research evidence. Chalmers et al. *Lancet* 2009). CRC, colorectal cancer.

There are serious concerns about methodology and study design affecting study quality. Poor reporting further complicates the assessment of results. Clinical usefulness of many diagnostic DNA methylation markers is questionable because they were only investigated in tissue. Even when assessed in bodily fluids, comparisons with the standard method (FIT) are rare, hampering definitive conclusions on their clinical utility (Figure 5).

Although extensively studied, only 3 DNA methylation markers (0.8%) are currently used in clinical practice. Although explorative studies on DNA methylation markers in small populations without validation can be valuable for scientific reasons, research aiming for clinical translation clearly needs a new approach.

First, as previously summarized, biomarker development should start with a consensus statement that a true clinical need is present (40). This is an important responsibility of researchers, research funders, and regulators (55); new research should only be initiated when research questions cannot be answered satisfactorily with available evidence (56). The main goal should be to improve the LoE of candidate biomarkers because this is crucial for clinical translation.

Second, the intended use of a biomarker should be clearly defined, as emphasized in guidelines (e.g., PRoBE) (51), and the biomarker needs to be evaluated in the target population with a design that fits the clinical application. Here, specimen and cohort choice plays an important role, especially in diseases such as CRC where only noninvasive diagnostic biomarkers are clinically relevant. To improve clinical practice, diagnostic biomarkers need to complement (or replace) existing diagnostic measures. Therefore, in CRC, comparison with FIT, preferably in the same study population, is a prerequisite. For this, definition of the performance measure is crucial and should be carefully considered (51,57).

Finally, reported data should support claims on the diagnostic potential of a marker and should be adequately reported to facilitate independent validation. This includes an adjustment of the reporting guidelines for biomarker studies and strict implementation of these guidelines by scientific journals.

Although the *Lancet* series on increasing value and reducing waste in biomedical research dates from 2014, there has unfortunately been little change in the DNA methylation marker field to avoid research waste. Although the drawbacks as stated in our review are applicable to diagnostic DNA methylation markers for CRC, they also apply to the broader biomarker research field. To stop this research waste in biomarker research, we need to raise the methodological bar and take into account some important considerations before designing biomarker studies to (i) improve reporting quality, (ii) facilitate external validation, (iii) facilitate meta-analyses, and (iv) improve LoE (Table 1). To reach this, a stronger focus on guidelines in initiating biomarker research is crucial. Only then we will be able to identify and develop biomarkers that can make a difference for patients.

**Study Highlights**

**WHAT IS KNOWN**

✓ Many candidate DNA methylation for the early detection of colorectal cancer have been suggested.
✓ Almost no early detection DNA methylation biomarkers are currently used in clinical practice, illustrating considerable research waste.
✓ There is a gap between biomarker discovery and clinical implementation.

**WHAT IS NEW HERE**

✓ An overview is provided of current evidence on early detection DNA methylation biomarkers for colorectal cancer.
✓ Methodological and technical issues hampering DNA methylation biomarker research are described.
✓ Recommendations are provided to reduce research waste and improve clinical translation of DNA methylation biomarkers.

**Table 1.** Recommendations for future DNA methylation biomarker studies

General
• Update reporting guidelines specifically for biomarker studies
**Before study design**
• Consensus statement clinical need
• Define intended biomarker use and select biomarkers accordingly
• Evaluate available evidence and current LoE
• Define study design to complement available evidence and add to LoE
• Define specimen and cohort to complement available evidence and facilitate external validation or meta-analyses
• Define the technical method to facilitate external validation and meta-analyses
• Define a suitable performance measure to facilitate external validation or meta-analyses
• Define the rationale for cutoff in quantitative analyses and/or define criteria for sensitivity and specificity a priori
**During/after study**
• Compare results with goldn standard methods (e.g., FIT) to complement available evidence
• Adhere to reporting guidelines to facilitate external validation or meta-analyses

FIT, fecal immunochemical test; LoE, level of evidence.

## CONFLICTS OF INTEREST

**Guarantor of the article:** Kim M. Smits, PhD.

**Specific author contributions:** M.v.E. and K.S. proposed the hypothesis and idea for the review. C.J.G.O. developed and did the initial literature search. Z.F. and C.J.G.O. reviewed studies for inclusion. Z.F., C.J.G.O., A.J.P.v.d.W., K.W., and K.M.S. did the data extraction and data checking. Z.F., C.J.G.O., A.J.P.v.d.W., and A.K. worked together on analyses and drafting the figures. Z.F., C.J.G.O., A.J.P.v.d.W., M.v.E., and K.S. developed the first draft of the manuscript. All authors reviewed and interpreted the results, edited the manuscript, and approved the final draft.

**Financial support:** Funding was provided by “Maag Lever Darm Stichting” (MLDS-project FP13-15) and a SU2C-DCS International Translational Cancer Research Dream Team Grant (Stand-Up-To-Cancer (SU2C)-AACR-DT1415,MEDOCC). The study funder had no role in study design, data collection, data analysis, data interpretation, or writing of the report.

**Potential competing interests:** Dr. van Engeland is co-founder and shareholder from Epify BV and MLA Diagnostics BV, and reports grants from MDxHealth, outside of the submitted work. In addition, Dr. van Engeland has a patent on DNA methylation markers for colorectal cancer detection.

## Supplementary Material
