# Supplementary material for: Lessons From a Systematic Literature Search on Diagnostic DNA Methylation Biomarkers for Colorectal Cancer: How to Increase Research Value and Decrease Research Waste?
Source: Clin Transl Gastroenterol. 2022 May 18;13(6):e00499. doi: 10.14309/ctg.0000000000000499 (PMC9236597; doi:10.14309/ctg.0000000000000499)
Supplement: Supplementary file 1 [file ct9-13-e00499-s001.docx]

**SUPPLEMENTARY MATERIAL**

**
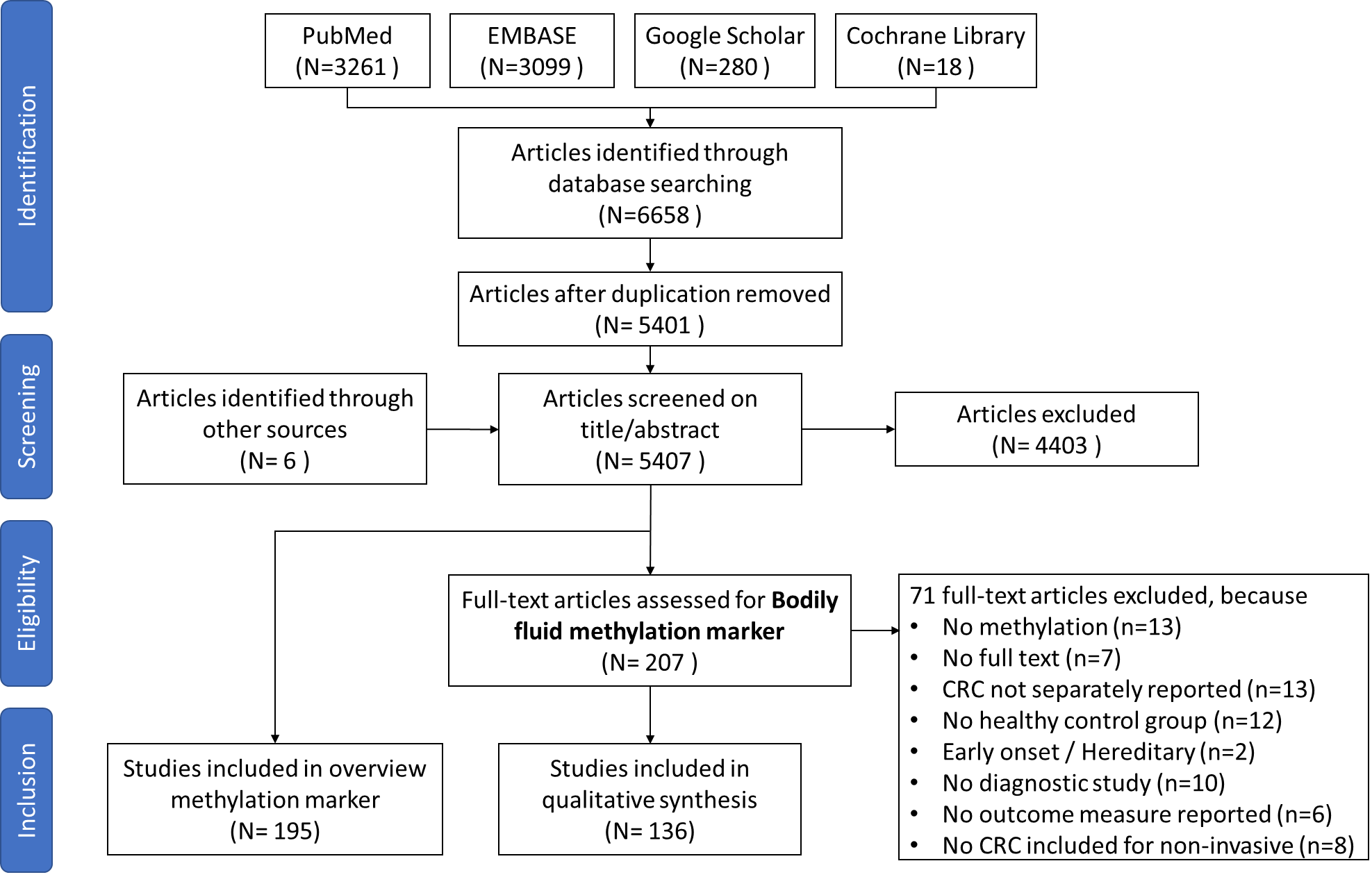
**

**Supplementary Figure 1**. Flowchart of the study identification process. A total of 136 articles reporting body fluid-based colorectal cancer DNA methylation markers were selected for qualitative assessment.

b

a

**Supplementary Figure 2**. Yearly identified (1985–2020) DNA methylation biomarkers for CRC detection in tissue **(a)** and body fluid **(b)** samples; The length of bars denotes total number of biomarkers investigated across all selected studies per year. Each bar is divided into two categories, with the orange color indicating the number of biomarkers newly identified in that year and the blue color representing the number of biomarkers discovered in previous years. (a)Total number of biomarkers identified in tissue samples per year. (b)Total number of biomarkers identified in bodily fluid samples per year.

**Supplementary Table 1**. Search Strategy for diagnostic methylation markers in colorectal cancer

|  | Pubmed | Embase | Cochrane Library | Google Scholar |
| --- | --- | --- | --- | --- |
| 1 | "methylation"[MeSH Terms] OR "methylation"[All Fields] OR hypermethylation[All Fields] OR hypomethylation[All Fields] OR promoter[All Fields] | ‘DNA methylation’ OR ‘hypermethylation’ OR ‘hypomethylation’ OR ‘methylation’ OR ‘promoter methylation’ | ‘methylation’ [MeSH Terms] | ‘DNA methylation’ |
| 2 | “colorectal neoplasms”[MeSH Terms] OR ((“colorectal”[All Fields] OR “rectal”[All Fields] OR “colon”[All Fields] OR “colonic”[All Fields] OR “sigmoid”[All Fields]) AND (“carcinoma”[All Fields] OR “cancer”[All Fields] OR “neoplasm”[All Fields] OR “tumor”[All Fields])) | ‘colorectal cancer’ OR ‘colon cancer’ OR ‘rectal cancer’ | ‘colorectal neoplasms’ [MeSH Terms] | ‘colorectal cancer’ |
| 3 | "diagnosis"[MeSH Terms] OR "diagnosis"[All Fields] OR "detection"[All Fields] | ‘diagnosis’ OR ‘detection’ | ‘diagnosis’ [MeSH Terms] | ‘diagnosis’ |
| 4 |  |  |  | ‘clinical test’ |
| Search | 1 and 2 and 3 | 1 and 2 and 3 | 1 and 2 and 3 | 1 and 2 and 3 and 4 |

**Supplementary Table 2**. Included references in the systematic review

| 1. | Abbaszadegan M.R., Tavasoli A., Velayati A., et al. Stool-based DNA testing, a new noninvasive method for colorectal cancer screening, the first report from Iran. World J Gastroenterol 2007, 13(10):1528-1533. |
| --- | --- |
| 2. | Ahlquist D.A., Taylor W.R., Mahoney D.W., et al. The stool DNA test is more accurate than the plasma septin 9 test in detecting colorectal neoplasia. Clin Gastroenterol Hepatol 2012, 10(3):272-277 e271. |
| 3. | Alizadeh Naini M., Kavousipour S., Hasanzarini M., et al. O6-Methyguanine-DNA Methyl Transferase (MGMT) Promoter Methylation in Serum DNA of Iranian Patients with Colorectal Cancer. Asian Pacific journal of cancer prevention : APJCP 2018, 19(5):1223-1227. |
| 4. | Ashoori H., Ghamarchehreh M.E., Tavallaei M., et al. Evaluation of the epigenetic biomarker bone morphogenic protein 3 for colorectal cancer diagnosis. Journal of Clinical and Diagnostic Research 2018, 12(11). |
| 5. | Babaei H., Mohammadi M., Salehi R. DNA methylation analysis of secreted frizzled-related protein 2 gene for the early detection of colorectal cancer in fecal DNA. Niger Med J 2016, 57(4):242-245. |
| 6. | Baek Y.H., Chang E., Kim Y.J., et al. Stool methylation-specific polymerase chain reaction assay for the detection of colorectal neoplasia in Korean patients. Dis Colon Rectum 2009, 52(8):1452-1459; discussion 1459-1463. |
| 7. | Bartak B.K., Kalmar A., Galamb O., et al. Blood Collection and Cell-Free DNA Isolation Methods Influence the Sensitivity of Liquid Biopsy Analysis for Colorectal Cancer Detection. Pathol Oncol Res 2018. |
| 8. | Bedin C., Enzo M.V., Del Bianco P., et al. Diagnostic and prognostic role of cell-free DNA testing for colorectal cancer patients. Int J Cancer 2017, 140(8):1888-1898. |
| 9. | Belshaw N.J., Elliott G.O., Williams E.A., et al. Use of DNA from human stools to detect aberrant CpG island methylation of genes implicated in colorectal cancer. Cancer Epidemiol Biomarkers Prev 2004, 13(9):1495-1501. |
| 10. | Bosch L.J., Oort F.A., Neerincx M., et al. DNA methylation of phosphatase and actin regulator 3 detects colorectal cancer in stool and complements FIT. Cancer Prev Res (Phila) 2012, 5(3):464-472. |
| 11. | Cassinotti E., Melson J., Liggett T., et al. DNA methylation patterns in blood of patients with colorectal cancer and adenomatous colorectal polyps. Int J Cancer 2012, 131(5):1153-1157. |
| 12. | Chang E., Park D.I., Kim Y.J., et al. Detection of colorectal neoplasm using promoter methylation of ITGA4, SFRP2, and p16 in stool samples: a preliminary report in Korean patients. Hepatogastroenterology 2010, 57(101):720-727. |
| 13. | Chen C.H., Yan S.L., Yang T.H., et al. The Relationship between the Methylated Septin-9 DNA Blood Test and Stool Occult Blood Test for Diagnosing Colorectal Cancer in Taiwanese People. J Clin Lab Anal 2017, 31(1). |
| 14. | Chen W.D., Han Z.J., Skoletsky J., et al. Detection in fecal DNA of colon cancer-specific methylation of the nonexpressed vimentin gene. J Natl Cancer Inst 2005, 97(15):1124-1132. |
| 15. | Church T.R., Wandell M., Lofton-Day C., et al. Prospective evaluation of methylated SEPT9 in plasma for detection of asymptomatic colorectal cancer. Gut 2014, 63(2):317-325. |
| 16. | deVos T., Tetzner R., Model F., et al. Circulating methylated SEPT9 DNA in plasma is a biomarker for colorectal cancer. Clin Chem 2009, 55(7):1337-1346. |
| 17. | Ebert M.P., Model F., Mooney S., et al. Aristaless-like homeobox-4 gene methylation is a potential marker for colorectal adenocarcinomas. Gastroenterology 2006, 131(5):1418-1430. |
| 18. | Elliott G.O., Johnson I.T., Scarll J., et al. Quantitative profiling of CpG island methylation in human stool for colorectal cancer detection. Int J Colorectal Dis 2013, 28(1):35-42. |
| 19. | Frattini M., Gallino G., Signoroni S., et al. Quantitative and qualitative characterization of plasma DNA identifies primary and recurrent colorectal cancer. Cancer Letters 2008, 263(2):170-181. |
| 20. | Fu B., Yan P., Zhang S., et al. Cell-Free Circulating Methylated SEPT9 for Noninvasive Diagnosis and Monitoring of Colorectal Cancer. Dis Markers 2018, 2018:6437104. |
| 21. | Gao Y. Leukocyte DNA methylation and colorectal cancer among male smokers. World Journal of Gastrointestinal Oncology 2012, 4(8). |
| 22. | Glockner S.C., Dhir M., Yi J.M., et al. Methylation of TFPI2 in stool DNA: a potential novel biomarker for the detection of colorectal cancer. Cancer Res 2009, 69(11):4691-4699. |
| 23. | Guo Q., Song Y., Zhang H., et al. Detection of hypermethylated fibrillin-1 in the stool samples of colorectal cancer patients. Med Oncol 2013, 30(4):695. |
| 24. | He C.G., Huang Q.Y., Chen L.S., et al. p33(ING1b) methylation in fecal DNA as a molecular screening tool for colorectal cancer and precancerous lesions. Oncol Lett 2014, 7(5):1639-1644. |
| 25. | He N., Song L., Kang Q., et al. The Pathological Features of Colorectal Cancer Determine the Detection Performance on Blood ctDNA. Technology in cancer research & treatment 2018, 17:1533033818791794. |
| 26. | Hellebrekers D.M., Lentjes M.H., van den Bosch S.M., et al. GATA4 and GATA5 are potential tumor suppressors and biomarkers in colorectal cancer. Clin Cancer Res 2009, 15(12):3990-3997. |
| 27. | Herbst A., Rahmig K., Stieber P., et al. Methylation of NEUROG1 in serum is a sensitive marker for the detection of early colorectal cancer. Am J Gastroenterol 2011, 106(6):1110-1118. |
| 28. | Huang Z., Li L., Wang J. Hypermethylation of SFRP2 as a potential marker for stool-based detection of colorectal cancer and precancerous lesions. Dig Dis Sci 2007, 52(9):2287-2291. |
| 29. | Huang Z.H., Li L.H., Yang F., et al. Detection of aberrant methylation in fecal DNA as a molecular screening tool for colorectal cancer and precancerous lesions. World J Gastroenterol 2007, 13(6):950-954. |
| 30. | Itzkowitz S., Brand R., Jandorf L., et al. A simplified, noninvasive stool DNA test for colorectal cancer detection. Am J Gastroenterol 2008, 103(11):2862-2870. |
| 31. | Itzkowitz S.H., Jandorf L., Brand R., et al. Improved fecal DNA test for colorectal cancer screening. Clin Gastroenterol Hepatol 2007, 5(1):111-117. |
| 32. | Jin P., Kang Q., Wang X., et al. Performance of a second-generation methylated SEPT9 test in detecting colorectal neoplasm. J Gastroenterol Hepatol 2015, 30(5):830-833. |
| 33. | Johnson D.A., Barclay R.L., Mergener K., et al. Plasma Septin9 versus fecal immunochemical testing for colorectal cancer screening: a prospective multicenter study. PLoS One 2014, 9(6):e98238. |
| 34. | Kalimutho M., Di Cecilia S., Del Vecchio Blanco G., et al. Epigenetically silenced miR-34b/c as a novel faecal-based screening marker for colorectal cancer. Br J Cancer 2011, 104(11):1770-1778. |
| 35. | Karam R.A., Zidan H.E., Abd Elrahman T.M., et al. Study of p16 promoter methylation in Egyptian colorectal cancer patients. Journal of Cellular Biochemistry 2018. |
| 36. | Kim M.S., Louwagie J., Carvalho B., et al. Promoter DNA methylation of oncostatin m receptor-beta as a novel diagnostic and therapeutic marker in colon cancer. PLoS One 2009, 4(8):e6555. |
| 37. | Kim S.J., Tae C.H., Hong S.N., et al. EYA4 Acts as a New Tumor Suppressor Gene in Colorectal Cancer. Mol Carcinog 2015, 54(12):1748-1757. |
| 38. | Kisiel J.B., Klepp P., Allawi H.T., et al. Analysis of DNA Methylation at Specific Loci in Stool Samples Detects Colorectal Cancer and High-Grade Dysplasia in Patients With Inflammatory Bowel Disease. Clin Gastroenterol Hepatol 2018. |
| 39. | Kisiel J.B., Yab T.C., Nazer Hussain F.T., et al. Stool DNA testing for the detection of colorectal neoplasia in patients with inflammatory bowel disease. Aliment Pharmacol Ther 2013, 37(5):546-554. |
| 40. | Kitkumthorn N., Tuangsintanakul T., Rattanatanyong P., et al. LINE-1 methylation in the peripheral blood mononuclear cells of cancer patients. Clin Chim Acta 2012, 413(9-10):869-874. |
| 41. | Kriegshauser G., Enko D., Zitt M., et al. Comparison of a prototype reverse hybridization assay and MethyLight for detection of SFRP2 promotor methylation in fecal DNA. Int J Biol Markers 2017, 32(4):e467-e470. |
| 42. | Leclerc D., Pham D.N., Levesque N., et al. Oncogenic role of PDK4 in human colon cancer cells. Br J Cancer 2017, 116(7):930-936. |
| 43. | Lee B.B., Lee E.J., Jung E.H., et al. Aberrant methylation of APC, MGMT, RASSF2A, and Wif-1 genes in plasma as a biomarker for early detection of colorectal cancer. Clin Cancer Res 2009, 15(19):6185-6191. |
| 44. | Lenhard K., Bommer G.T., Asutay S., et al. Analysis of promoter methylation in stool: a novel method for the detection of colorectal cancer. Clin Gastroenterol Hepatol 2005, 3(2):142-149. |
| 45. | Leung W.K., To K.F., Man E.P., et al. Detection of epigenetic changes in fecal DNA as a molecular screening test for colorectal cancer: a feasibility study. Clin Chem 2004, 50(11):2179-2182. |
| 46. | Leung W.K., To K.F., Man E.P., et al. Quantitative detection of promoter hypermethylation in multiple genes in the serum of patients with colorectal cancer. Am J Gastroenterol 2005, 100(10):2274-2279. |
| 47. | Leung W.K., To K.-F., Man E.P.S., et al. Detection of Hypermethylated DNA or Cyclooxygenase-2 Messenger RNA in Fecal Samples of Patients With Colorectal Cancer or Polyps. The American Journal of Gastroenterology 2007, 102(5):1070-1076. |
| 48. | Li W.H., Zhang H., Guo Q., et al. Detection of SNCA and FBN1 methylation in the stool as a biomarker for colorectal cancer. Dis Markers 2015, 2015:657570. |
| 49. | Liu Y., Tham C.K., Ong S.Y., et al. Serum methylation levels of TAC1. SEPT9 and EYA4 as diagnostic markers for early colorectal cancers: a pilot study. Biomarkers 2013, 18(5):399-405. |
| 50. | Lofton-Day C., Model F., Devos T., et al. DNA methylation biomarkers for blood-based colorectal cancer screening. Clin Chem 2008, 54(2):414-423. |
| 51. | Lu H., Huang S., Zhang X., et al. DNA methylation analysis of SFRP2, GATA4/5, NDRG4 and VIM for the detection of colorectal cancer in fecal DNA. Oncol Lett 2014, 8(4):1751-1756. |
| 52. | Marsit C.J., Lange C.P.E., Campan M., et al. Genome-Scale Discovery of DNA-Methylation Biomarkers for Blood-Based Detection of Colorectal Cancer. PLoS ONE 2012, 7(11). |
| 53. | Mayor R., Casadome L., Azuara D., et al. Long-range epigenetic silencing at 2q14.2 affects most human colorectal cancers and may have application as a non-invasive biomarker of disease. Br J Cancer 2009, 100(10):1534-1539. |
| 54. | Melotte V., Lentjes M.H., van den Bosch S.M., et al. N-Myc downstream-regulated gene 4 (NDRG4): a candidate tumor suppressor gene and potential biomarker for colorectal cancer. J Natl Cancer Inst 2009, 101(13):916-927. |
| 55. | Melotte V., Yi J.M., Lentjes M.H., et al. Spectrin repeat containing nuclear envelope 1 and forkhead box protein E1 are promising markers for the detection of colorectal cancer in blood. Cancer Prev Res (Phila) 2015, 8(2):157-164. |
| 56. | Melson J., Li Y., Cassinotti E., et al. Commonality and differences of methylation signatures in the plasma of patients with pancreatic cancer and colorectal cancer. Int J Cancer 2014, 134(11):2656-2662. |
| 57. | Miotto E., Sabbioni S., Veronese A., et al. Frequent aberrant methylation of the CDH4 gene promoter in human colorectal and gastric cancer. Cancer Res 2004, 64(22):8156-8159. |
| 58. | Mitchell S.M., Ho T., Brown G.S., et al. Evaluation of Methylation Biomarkers for Detection of Circulating Tumor DNA and Application to Colorectal Cancer. Genes (Basel) 2016, 7(12). |
| 59. | Müller H.M., Oberwalder M., Fiegl H., et al. Methylation changes in faecal DNA: a marker for colorectal cancer screening? The Lancet 2004, 363(9417):1283-1285. |
| 60. | Nagai Y., Sunami E., Yamamoto Y., et al. LINE-1 hypomethylation status of circulating cell-free DNA in plasma as a biomarker for colorectal cancer. Oncotarget 2017, 8(7):11906-11916. |
| 61. | Nagasaka T., Tanaka N., Cullings H.M., et al. Analysis of fecal DNA methylation to detect gastrointestinal neoplasia. J Natl Cancer Inst 2009, 101(18):1244-1258. |
| 62. | Najbauer J., Grützmann R., Molnar B., et al. Sensitive Detection of Colorectal Cancer in Peripheral Blood by Septin 9 DNA Methylation Assay. PLoS ONE 2008, 3(11). |
| 63. | Nan H., Giovannucci E.L., Wu K., et al. Pre-diagnostic leukocyte genomic DNA methylation and the risk of colorectal cancer in women. PLoS One 2013, 8(4):e59455. |
| 64. | Nishio M., Sakakura C., Nagata T., et al. RUNX3 promoter methylation in colorectal cancer: its relationship with microsatellite instability and its suitability as a novel serum tumor marker. Anticancer Res 2010, 30(7):2673-2682. |
| 65. | Niu F., Wen J., Fu X., et al. Stool DNA Test of Methylated Syndecan-2 for the Early Detection of Colorectal Neoplasia. Cancer Epidemiology Biomarkers & Prevention 2017, 26(9):1411-1419. |
| 66. | Nunes S.P., Moreira-Barbosa C., Salta S., et al. Cell-free DNA methylation of selected genes allows for early detection of the major cancers in women. Cancers 2018, 10(10):357. |
| 67. | Oh T., Kim N., Moon Y., et al. Genome-wide identification and validation of a novel methylation biomarker, SDC2, for blood-based detection of colorectal cancer. J Mol Diagn 2013, 15(4):498-507. |
| 68. | Oh T.J., Oh H.I., Seo Y.Y., et al. Feasibility of quantifying SDC2 methylation in stool DNA for early detection of colorectal cancer. Clin Epigenetics 2017, 9:126. |
| 69. | Orntoft M.B., Nielsen H.J., Orntoft T.F., et al. Performance of the colorectal cancer screening marker Sept9 is influenced by age, diabetes and arthritis: a nested case-control study. BMC Cancer 2015, 15:819. |
| 70. | Pack S.C., Kim H.R., Lim S.W., et al. Usefulness of plasma epigenetic changes of five major genes involved in the pathogenesis of colorectal cancer. Int J Colorectal Dis 2013, 28(1):139-147. |
| 71. | Park S.K., Baek H.L., Yu J., et al. Is methylation analysis of SFRP2, TFPI2, NDRG4, and BMP3 promoters suitable for colorectal cancer screening in the Korean population? Intest Res 2017, 15(4):495-501. |
| 72. | Pedersen S.K., Baker R.T., McEvoy A., et al. A two-gene blood test for methylated DNA sensitive for colorectal cancer. PLoS One 2015, 10(4):e0125041. |
| 73. | Pedersen S.K., Mitchell S.M., Graham L.D., et al. CAHM, a long non-coding RNA gene hypermethylated in colorectal neoplasia. Epigenetics 2014, 9(8):1071-1082. |
| 74. | Pedersen S.K., Symonds E.L., Baker R.T., et al. Evaluation of an assay for methylated BCAT1 and IKZF1 in plasma for detection of colorectal neoplasia. BMC Cancer 2015, 15:654. |
| 75. | Potter N.T., Hurban P., White M.N., et al. Validation of a real-time PCR-based qualitative assay for the detection of methylated SEPT9 DNA in human plasma. Clin Chem 2014, 60(9):1183-1191. |
| 76. | Rasmussen S.L., Krarup H.B., Sunesen K.G., et al. Hypermethylated DNA, a circulating biomarker for colorectal cancer detection. PLoS One 2017, 12(7):e0180809. |
| 77. | Ravegnini G., Zolezzi Moraga J.M., Maffei F., et al. Simultaneous Analysis of SEPT9 Promoter Methylation Status, Micronuclei Frequency, and Folate-Related Gene Polymorphisms: The Potential for a Novel Blood-Based Colorectal Cancer Biomarker. Int J Mol Sci 2015, 16(12):28486-28497. |
| 78. | Rezvani N., Alibakhshi R., Vaisi-Raygani A., et al. Detection of SPG20 gene promoter-methylated DNA, as a novel epigenetic biomarker, in plasma for colorectal cancer diagnosis using the MethyLight method. Oncol Lett 2017, 13(5):3277-3284. |
| 79. | Roperch J.P., Incitti R., Forbin S., et al. Aberrant methylation of NPY, PENK, and WIF1 as a promising marker for blood-based diagnosis of colorectal cancer. BMC Cancer 2013, 13:566. |
| 80. | Sabbioni S., Miotto E., Veronese A., et al. Multigene methylation analysis of gastrointestinal tumors: TPEF emerges as a frequent tumor-specific aberrantly methylated marker that can be detected in peripheral blood. Mol Diagn 2003, 7(3-4):201-207. |
| 81. | Sakamoto J., Fujiya M., Okamoto K., et al. Immunoprecipitation of nucleosomal DNA is a novel procedure to improve the sensitivity of serum screening for the p16 hypermethylation associated with colon cancer. Cancer Epidemiol 2010, 34(2):194-199. |
| 82. | Salehi R., Atapour N., Vatandoust N., et al. Methylation pattern of ALX4 gene promoter as a potential biomarker for blood-based early detection of colorectal cancer. Adv Biomed Res 2015, 4:252. |
| 83. | Salehi R., Mohammadi M., Emami M.H., et al. Methylation pattern of SFRP1 promoter in stool sample is a potential marker for early detection of colorectal cancer. Adv Biomed Res 2012, 1:87. |
| 84. | Shirahata A., Hibi K. Serum vimentin methylation as a potential marker for colorectal cancer. Anticancer Res 2014, 34(8):4121-4125. |
| 85. | Song B.P., Jain S., Lin S.Y., et al. Detection of hypermethylated vimentin in urine of patients with colorectal cancer. J Mol Diagn 2012, 14(2):112-119. |
| 86. | Song L., Wang J., Wang H., et al. The quantitative profiling of blood mSEPT9 determines the detection performance on colorectal tumors. Epigenomics 2018, 10(12):1569-1583. |
| 87. | Suehiro Y., Hashimoto S., Higaki S., et al. Blood free-circulating DNA testing by highly sensitive methylation assay to diagnose colorectal neoplasias. Oncotarget 2018, 9(24):16974-16987. |
| 88. | Suehiro Y., Zhang Y., Hashimoto S., et al. Highly sensitive faecal DNA testing of TWIST1 methylation in combination with faecal immunochemical test for haemoglobin is a promising marker for detection of colorectal neoplasia. Annals of Clinical Biochemistry 2018, 55(1):59-68. |
| 89. | Symonds E.L., Pedersen S.K., Baker R.T., et al. A Blood Test for Methylated BCAT1 and IKZF1 vs. a Fecal Immunochemical Test for Detection of Colorectal Neoplasia. Clin Transl Gastroenterol 2016, 7:e137. |
| 90. | Takane K., Midorikawa Y., Yagi K., et al. Aberrant promoter methylation of PPP1R3C and EFHD1 in plasma of colorectal cancer patients. Cancer Med 2014, 3(5):1235-1245. |
| 91. | Tang D., Liu J., Wang D.R., et al. Diagnostic and prognostic value of the methylation status of secreted frizzled-related protein 2 in colorectal cancer. Clin Invest Med 2011, 34(2):E88-95. |
| 92. | Tanzer M., Balluff B., Distler J., et al. Performance of epigenetic markers SEPT9 and ALX4 in plasma for detection of colorectal precancerous lesions. PLoS One 2010, 5(2):e9061. |
| 93. | Toth K., Sipos F., Kalmar A., et al. Detection of methylated SEPT9 in plasma is a reliable screening method for both left- and right-sided colon cancers. PLoS One 2012, 7(9):e46000. |
| 94. | Toth K., Wasserkort R., Sipos F., et al. Detection of methylated septin 9 in tissue and plasma of colorectal patients with neoplasia and the relationship to the amount of circulating cell-free DNA. PLoS One 2014, 9(12):e115415. |
| 95. | Wang D.R., Tang D. Hypermethylated SFRP2 gene in fecal DNA is a high potential biomarker for colorectal cancer noninvasive screening. World J Gastroenterol 2008, 14(4):524-531. |
| 96. | Wang Y.C., Yu Z.H., Liu C., et al. Detection of RASSF1A promoter hypermethylation in serum from gastric and colorectal adenocarcinoma patients. World J Gastroenterol 2008, 14(19):3074-3080. |
| 97. | Warren J.D., Xiong W., Bunker A.M., et al. Septin 9 methylated DNA is a sensitive and specific blood test for colorectal cancer. BMC Med 2011, 9:133. |
| 98. | Wu D., Zhou G., Jin P., et al. Detection of Colorectal Cancer Using a Simplified SEPT9 Gene Methylation Assay Is a Reliable Method for Opportunistic Screening. J Mol Diagn 2016, 18(4):535-545. |
| 99. | Wu P.P., Zou J.H., Tang R.N., et al. Detection and Clinical Significance of DLC1 Gene Methylation in Serum DNA from Colorectal Cancer Patients. Chin J Cancer Res 2011, 23(4):283-287. |
| 100. | Wu X.D., Song Y.C., Cao P.L., et al. Detection of miR-34a and miR-34b/c in stool sample as potential screening biomarkers for noninvasive diagnosis of colorectal cancer. Med Oncol 2014, 31(4):894. |
| 101. | Xiao W., Zhao H., Dong W., et al. Quantitative detection of methylated NDRG4 gene as a candidate biomarker for diagnosis of colorectal cancer. Oncol Lett 2015, 9(3):1383-1387. |
| 102. | Xiao Z., Li B., Wang G., et al. Validation of methylation-sensitive high-resolution melting (MS-HRM) for the detection of stool DNA methylation in colorectal neoplasms. Clin Chim Acta 2014, 431:154-163. |
| 103. | Xie L., Jiang X., Li Q., et al. Diagnostic value of methylated Septin9 for colorectal cancer detection. Frontiers in Oncology 2018, 8(JUL):247. |
| 104. | Zhang H., Song Y.C., Dang C.X. Detection of hypermethylated spastic paraplegia-20 in stool samples of patients with colorectal cancer. Int J Med Sci 2013, 10(3):230-234. |
| 105. | Zhang J., Yang S., Xie Y., et al. Detection of methylated tissue factor pathway inhibitor 2 and human long DNA in fecal samples of patients with colorectal cancer in China. Cancer Epidemiol 2012, 36(1):73-77. |
| 106. | Zhang T., Cui G., Yao Y.L., et al. Value of CNRIP1 promoter methylation in colorectal cancer screening and prognosis assessment and its influence on the activity of cancer cells. Arch Med Sci 2017, 13(6):1281-1294. |
| 107. | Zhang W., Bauer M., Croner R.S., et al. DNA Stool Test for Colorectal Cancer: Hypermethylation of the Secreted Frizzled-Related Protein-1 Gene. Diseases of the Colon & Rectum 2007, 50(10):1618-1627. |
| 108. | Zhang X., Song Y.F., Lu H.N., et al. Combined detection of plasma GATA5 and SFRP2 methylation is a valid noninvasive biomarker for colorectal cancer and adenomas. World J Gastroenterol 2015, 21(9):2629-2637. |
| 109. | Zhou D., Tang W., Su G., et al. PCDH18 is frequently inactivated by promoter methylation in colorectal cancer. Sci Rep 2017, 7(1):2819. |
| 110 | Chen J, Sun H, Tang W, et al. DNA methylation biomarkers in stool for early screening of colorectal cancer. J Cancer. 2019;10(21):5264-71. |
| 111 | Chen Y, Wang ZZ, Zhao GD, et al. Performance of a Novel Blood-Based Early Colorectal Cancer Screening Assay in Remaining Serum after the Blood Biochemical Test. Dis Markers. 2019; Article ID 5232780, 6 pages, 2019. |
| 112 | Jensen SO, Ogaard N, Orntoft MW, et al. Novel DNA methylation biomarkers show high sensitivity and specificity for blood-based detection of colorectal cancer-a clinical biomarker discovery and validation study. Clin Epigenetics. 2019;11(1):158. |
| 113 | Li H, Wang Z, Zhao G, et al. Performance of a MethyLight assay for methylated SFRP2 DNA detection in colorectal cancer tissue and serum. Int J Biol Markers. 2019;34(1):54-9. |
| 114 | Ma ZY, Law WL, Ng EKO, et al. Methylated Septin 9 and Carcinoembryonic Antigen for Serological Diagnosis and Monitoring of Patients with Colorectal Cancer After Surgery. Sci Rep. 2019;9(1):10326. |
| 115 | Pakbaz B, Jabinin R, Soltani N, et al. Quantitative study of vimentin gene methylation in stool samples for colorectal cancer screening. J Adv Pharm Technol Res. 2019;10(3):121-5. |
| 116 | Pasha HF, Radwan MI, Yehia AM, et al. Circulating methylated RUNX3 and SFRP1 genes as a noninvasive panel for early detection of colorectal cancer. Eur J Gastroenterol Hepatol. 2019;31(11):1342-9. |
| 117 | Picardo F, Romanelli A, Muinelo-Romay L, et al. Diagnostic and Prognostic Value of B4GALT1 Hypermethylation and Its Clinical Significance as a Novel Circulating Cell-Free DNA Biomarker in Colorectal Cancer. Cancers (Basel). 2019;11(10). |
| 118 | Salama RH, Sayed ZEA, Ashmawy AM, et al. Interrelations of Apoptotic and Cellular Senescence Genes Methylation in Inflammatory Bowel Disease Subtypes and Colorectal Carcinoma in Egyptians Patients. Appl Biochem Biotechnol. 2019;189(1):330-43. |
| 119 | Sun J, Fei F, Zhang M, Li Y, et al. The role of (m)SEPT9 in screening, diagnosis, and recurrence monitoring of colorectal cancer. BMC Cancer. 2019;19(1):450. |
| 120 | Sun M, Liu J, Hu H, et al. A novel panel of stool-based DNA biomarkers for early screening of colorectal neoplasms in a Chinese population. J Cancer Res Clin Oncol. 2019;145(10):2423-32. |
| 121 | Zhao G, Li H, Yang Z, et al. Multiplex methylated DNA testing in plasma with high sensitivity and specificity for colorectal cancer screening. Cancer Med. 2019;8(12):5619-28. |
| 122 | Bagheri H, Mosallaei M, Bagherpour B, et al. TFPI2 and NDRG4 gene promoter methylation analysis in peripheral blood mononuclear cells are novel epigenetic noninvasive biomarkers for colorectal cancer diagnosis. J Gene Med. 2020;22(8):e3189. |
| 123 | Bhat ZI, Kumar B, Bansal S, et al. Association of PARK2 promoter polymorphisms and methylation with colorectal cancer in North Indian population. Gene. 2019;682:25-32. |
| 124 | Bi H, Liu Y, Pu R, et al. CHST7 Gene Methylation and Sex-Specific Effects on Colorectal Cancer Risk. Dig Dis Sci. 2019;64(8):2158-66. |
| 125 | Constancio V, Nunes SP, Moreira-Barbosa C, et al. Early detection of the major male cancer types in blood-based liquid biopsies using a DNA methylation panel. Clin Epigenetics. 2019;11(1):175. |
| 126 | Kerachian MA, Javadmanesh A, Azghandi M, et al. Crosstalk between DNA methylation and gene expression in colorectal cancer, a potential plasma biomarker for tracing this tumor. Sci Rep. 2020;10(1):2813. |
| 127 | Yong-Suk Kim, Song-Hak Kim, Myong-Nam Kim, et al. SFRP2 Promoter Methylation Analysis in Tumor Tissue, Stool, and Plasma DNA of Patients with Colorectal Cancer. Arch Intern Med Res. 2019;2(3):34-9. |
| 128 | Liu X, Wen J, Li C, et al. High-Yield Methylation Markers for Stool-Based Detection of Colorectal Cancer. Dig Dis Sci. 2020;65(6):1710-9. |
| 129 | Liu Y, Zhao G, Miao J, et al. Performance Comparison Between Plasma and Stool Methylated SEPT9 Tests for Detecting Colorectal Cancer. Front Genet. 2020;11:324. |
| 130 | Luo H, Zhao Q, Wei W, et al. Circulating tumor DNA methylation profiles enable early diagnosis, prognosis prediction, and screening for colorectal cancer. Sci Transl Med. 2020;12(524). |
| 131 | Suehiro Y, Hashimoto S, Goto A, et al. Fecal DNA Testing of TWIST1 Methylation Identifies Patients With Advanced Colorectal Adenoma Missed by Fecal Immunochemical Test for Hemoglobin. Clin Transl Gastroenterol. 2020;11(6):e00176. |
| 132 | Vega-Benedetti AF, Loi E, Moi L, et al. Colorectal Cancer Early Detection in Stool Samples Tracing CpG Islands Methylation Alterations Affecting Gene Expression. Int J Mol Sci. 2020;21(12). |
| 133 | Yang C, Wu W, Yang Y, et al. Multitarget stool DNA test compared with fecal occult blood test for colorectal cancer screening. Oncol Lett. 2020;20(2):1193-200. |
| 134 | Zhao G, Ma Y, Li H, et al. A novel plasma based early colorectal cancer screening assay base on methylated SDC2 and SFRP2. Clin Chim Acta. 2020;503:84-9. |
| 135 | Zhao G, Liu X, Liu Y, et al. Aberrant DNA Methylation of SEPT9 and SDC2 in Stool Specimens as an Integrated Biomarker for Colorectal Cancer Early Detection. Front Genet. 2020;11:643. |
| 136 | Han YD, Oh TJ, Chung TH, et al. Early detection of colorectal cancer based on presence of methylated syndecan-2 (SDC2) in stool DNA. Clin Epigenetics. 2019;11(1):51. |

**Supplementary Table 3**. STARD checklist, adapted for this study

| **Section** | **No** | **Item** | **Interpretation and scoring** |
| --- | --- | --- | --- |
| **Title or abstract** | | | |
|  | 1 | Identification as a study of diagnostic accuracy using at least one measure of accuracy (such as sensitivity, specificity, predictive values, or AUC) |  |
| **Abstract** | | | |
|  | 2 | Structured summary of study design, methods, results, and conclusions (for specific guidance, see STARD for Abstracts) |  |
| **Introduction** | | | |
|  | 3 | Scientific and clinical background, including the intended use and clinical role of the index test | Intended use or clinical role not mentioned = 0.5 |
|  | 4 | Study objectives and hypotheses | Also 1.0 point if only objectives are mentioned |
| **Methods** | | | |
| Study design | 5 | Whether data collection was planned before the index test and reference standard were performed (prospective study) or after (retrospective study) | If described: 1.0 point, if you can implicitly conclude it from text: 0.5 point |
| Participants | 6 | Eligibility criteria |  |
|  | 7 | On what basis potentially eligible participants were identified (such as symptoms, results from previous tests, inclusion in registry) |  |
|  | 8 | Where and when potentially eligible participants were identified (setting, location, and dates) |  |
|  | 9 | Whether participants formed a consecutive, random, or convenience series |  |
| Test methods | 10a | Index test, in sufficient detail to allow replication | In case the article referred to another article for primer and probe sequences, 0.5 point was awarded. |
|  | 10b | Reference standard, in sufficient detail to allow replication | Excluded. |
|  | 11 | Rationale for choosing the reference standard (if alternatives exist) | Excluded. |
|  | 12a | Definition of and rationale for test positivity cut-offs or result categories of the index test, distinguishing pre-specified from exploratory | (Q-MSP should usually define cut-off) |
|  | 12b | Definition of and rationale for test positivity cut-offs or result categories of the reference standard, distinguishing pre-specified from exploratory | Excluded. |
|  | 13a | Whether clinical information and reference standard results were available to the performers or readers of the index test |  |
|  | 13b | Whether clinical information and index test results were available to the assessors of the reference standard | Excluded. |
| Analysis | 14 | Methods for estimating or comparing measures of diagnostic accuracy | Only frequencies = 0.5 points, if sens + spec are mentioned = 1.0 point. |
|  | 15 | How indeterminate index test or reference standard results were handled |  |
|  | 16 | How missing data on the index test and reference standard were handled | Excluded. |
|  | 17 | Any analyses of variability in diagnostic accuracy, distinguishing pre-specified from exploratory | Excluded. |
|  | 18 | Intended sample size and how it was determined |  |
| **Results** | | | |
| Participants | 19 | Flow of participants, using a diagram | Excluded. |
|  | 20 | Baseline demographic and clinical characteristics of participants | If characteristics are not summarized, thus individual patient samples are described (often in supplement) 0.5 points. |
|  | 21a | Distribution of severity of disease in those with the target condition |  |
|  | 21b | Distribution of alternative diagnoses in those without the target condition | Excluded. |
|  | 22 | Time interval and any clinical interventions between index test and reference standard | Excluded. |
| Test results | 23 | Cross tabulation of the index test results (or their distribution) by the results of the reference standard | Cross tabulation or distribution of methylation marker for healthy/CRC = 1.0 point |
|  | 24 | Estimates of diagnostic accuracy and their precision (such as 95% confidence intervals) | Precision for estimates of diagnostic accuracy (such as 95% confidence intervals) are reported = 1.0 point  only frequencies with CI/SD/SEM =0.5 points  no CI's = 0 |
|  | 25 | Any adverse events from performing the index test or the reference standard | Excluded. |
| **Discussion** | | | |
|  | 26 | Study limitations, including sources of potential bias, statistical uncertainty, and generalisability | In case only one limitation was mentioned or limitations were mentioned without explanation, 0.5 point was awarded.  No limitations mentioned = 0 point |
|  | 27 | Implications for practice, including the intended use and clinical role of the index test |  |
| **Other information** | | | |
|  | 28 | Registration number and name of registry | Excluded. |
|  | 29 | Where the full study protocol can be accessed | Excluded. |
|  | 30 | Sources of funding and other support; role of funders |  |

**Supplementary Table 5.** Study characteristics of the studies regarding diagnostic CRC markers in bodily fluids.

| **Study characteristics** | | | | | **Cases** | | | | | **Controls** | | | | **Diagnostic performance** | | | | | **Experimental methods** | |
| --- | --- | --- | --- | --- | --- | --- | --- | --- | --- | --- | --- | --- | --- | --- | --- | --- | --- | --- | --- | --- |
|  | Design | Marker | Sample | Collection period | N | Age, mean±SD  (range) | TNM/Dukes stage | Tumor location | Methylation,% (CI) | Type | N | Age, mean±SD  (range) | Methylation %  (CI) | Se,%  (CI) | Sp,%  (CI) | AUC,%  (CI) | PPV,%  (CI) | NPV,%  (CI) | DNA isolation | Methylation method |
| Abbaszadegan 2007 [1] | P. | p16 | stool |  | 21 | 59.2 ± 13.3 (34- 83) | B1 14%,  B2 52%,  C2 24%,  UN 10% | 40% distal  60% proximal | 20 |  | 20 |  | 0 | 20 | 100 |  |  |  | Phenol / chloroform | MSP |
| Ahlquist 2012 [2] | R. | *SEPT9* | plasma |  | 52 | 69  (61-75) | I 23%,  II 23%,  III 27%,  IV 27% | 50% distal  50% proximal | 60  (41-77) | NC | 49 | 63  (52-71) | 27 | 60  (41-77) | 73  (58-85) |  |  |  | Exact Sciences | Exact Sciences |
| Naini 2018 [3] | CC | *MGMT* | serum | 2013-2014 | 30 |  | I 20%,  II 56.7%,  III 20%,  IV 3.3% |  |  | NC | 40 |  |  | 90 | 100 |  |  |  | EZ DNA  Methylation Kit | MSP |
| Ashoori 2018 [4] | CC | *BMP3* | plasma | 2011-2014 | 59 | 62.2 |  |  |  | NED | 37 | 60.6 |  | 66 | 76 |  |  |  | QIAamp DNA Blood Mini Kit | MSP |
| Babaei 2016 [5] | P | *SFRP2* | stool |  | 20 | 58 |  |  | 60 | H | 58 |  | 8 | 60 | 92 |  |  |  | QIAamp DNA Stool Mini Kit | MSP |
| Baek 2009 [6] | P | *MLH1* | stool | 2007-2008 | 60 | 61.4±8.1 | I-II 58.3%  III-IV 41.7% | 88.3% distal  11.7% proximal | 30 | NC | 37 | 58.8±10.7 |  | 30 |  |  |  |  | QIAamp DNA Stool Mini Kit | MSP |
|  |  | *MGMT* |  |  |  |  |  |  | 51.7 |  |  |  |  | 51.7 |  |  |  |  |  |  |
|  |  | *VIM* |  |  |  |  |  |  | 38.3 |  |  |  |  | 38.3 |  |  |  |  |  |  |
|  |  | *MLH1, Vimentin, MGMT panel* |  |  |  |  |  |  | 75 |  |  |  | 13.5 | 75 | 86.5 |  |  |  |  |  |
| Bartak 2017 [7] | P | *PRIMA1* | plasma |  | 47 |  | A-B 57.4%  C-D 42.6% |  | 80.9 |  | 37 |  | 27 | 57.4 | 100 | 82.2  (73.8-90.7) |  |  | EZ DNA methylation kit | MethyLight |
|  |  | *SDC2* |  |  |  |  |  |  | 89.4 |  |  |  | 2.7 | 87.2 | 100 | 93  (87.1-98.8) |  |  |  |  |
|  |  | *SFRP1* |  |  |  |  |  |  | 85.1 |  |  |  | 18.9 | 80.9 | 83.8 | 86.9  (79.5-94.2) |  |  |  |  |
|  |  | *SFRP2* |  |  |  |  |  |  | 72.3 |  |  |  | 10.8 | 63.8 | 97.3 | 86.3  (78.9-9.37) |  |  |  |  |
|  |  | *SFRP1 + SFRP2 + SDC2 + PRIMA1* |  |  |  |  |  |  |  |  |  |  |  | 91.5 | 97.3 | 97.8  (95.4-100) |  |  |  |  |
| Bedin 2017 [8] | P | *OSMR* | plasma |  | 70 | 67  (61-73) | I 16%  II 21%  III 42%  IV 21% | 70% distal  30% proximal | 44 | H | 36 | 66  (59-73) | 14 | 44.3 | 86.1 | 69 | 86.1 | 44.3 | QIAamp DNA Mini Kit | Q-PCR |
|  |  | *SFRP1* |  |  |  |  |  |  | 63 |  |  |  | 8 | 62.9 | 91.7 | 79.5 | 93.6 | 55.9 |  |  |
|  |  | *OSMR + SFRP1* |  |  |  |  |  |  | 67 |  |  |  | 19 | 67.1 | 80.6 |  | 87 | 55.8 |  |  |
| Belshaw 2004 [9] | P | *APC* | stool |  | 12 |  |  |  | 1.6 | H | 21 |  | 1.7 |  |  |  |  |  | QIAamp DNA Stool mini kit | MSP and COBRA |
|  |  | *HPP1* |  |  |  |  |  |  | 1.8 |  |  |  | 1.5 |  |  |  |  |  |  |  |
|  |  | *MGMT* |  |  |  |  |  |  | 14.1 |  |  |  | 20.4 |  |  |  |  |  |  |  |
|  |  | *MLH1* |  |  |  |  |  |  | 0.7 |  |  |  | 0.6 |  |  |  |  |  |  |  |
|  |  | *p16ink4a* |  |  |  |  |  |  | 2.1 |  |  |  | 1.1 |  |  |  |  |  |  |  |
|  |  | *ESR1 (+164)* |  |  |  |  |  |  | 44.2 |  |  |  | 42.8 |  |  |  |  |  |  |  |
|  |  | *ESR1 (+223)* |  |  |  |  |  |  | 33.6 |  |  |  | 36.8 |  |  |  |  |  |  |  |
|  |  | *ESR1 (+313)* |  |  |  |  |  |  | 39.1 |  |  |  | 38.5 |  |  |  |  |  |  |  |
| Bosch 2012 [10] |  | *GATA4* | stool |  | 21 | 69.7±8.6 | I 23%,  II 36%,  III 30%,  IV 9%,  UN 2% |  |  | H | 60 | 55.3±10.4 |  | 57 | 94 | 76  (62-90) |  |  | QIAamp DNA Stool mini kit | qMSP |
|  |  | *OSMR* |  |  |  |  |  |  |  |  |  |  |  | 29 | 94 | 60  (45-75) |  |  |  |  |
|  |  | *PHACTR3* |  |  | 22 |  |  |  |  |  | 66 |  |  | 55  (33-75) | 95  (87-98) | 78  (64-91) |  |  |  |  |
|  |  | *PHACTR3 + OSMR + GATA4* |  |  |  |  |  |  |  |  |  |  |  | 62 | 87 |  |  |  |  |  |
|  |  | *GATA4* | stool |  | 44 | 71±9.3 | I 23%,  II 36%,  III 30%,  IV 9%,  UN 2% |  |  | H | 30 | 51.8±9.9 |  | 39 | 93 |  |  |  | QIAamp DNA Stool mini kit | qMSP |
|  |  | *OSMR* |  |  |  |  |  |  |  |  |  |  |  | 43 | 90 |  |  |  |  |  |
| Bosch 2012 [10] |  | *PHACTR3* |  |  |  |  |  |  |  |  |  |  |  | 66  (50-79) | 100  (86-100) | 87  (79-95) |  |  |  |  |
|  |  | *PHACTR3 + OSMR + GATA4* |  |  |  |  |  |  |  |  |  |  |  | 68 | 83 |  |  |  |  |  |
| Cassinotti 2012 [11] | P | *CYCD2* | plasma |  | 30 | 68.3  (49-85) | I 37%,  II 63% | 73% distal,  27% proximal | 96.7 | H | 30 | 61.2  (40-80) | 63.3 | 97 | 37 |  |  |  | DNAzol BD + proteinase K | MethDet 56 |
|  |  | *HIC1* |  |  |  |  |  |  | 63.3 |  |  |  | 6.7 | 63 | 93 |  |  |  |  |  |
|  |  | *PAX5* |  |  |  |  |  |  | 86.7 |  |  |  | 56.7 | 87 | 43 |  |  |  |  |  |
|  |  | *RASSF1A* |  |  |  |  |  |  | 93.3 |  |  |  | 46.7 | 93 | 53 |  |  |  |  |  |
|  |  | *RB1* |  |  |  |  |  |  | 90 |  |  |  | 46.7 | 90 | 53 |  |  |  |  |  |
|  |  | *SRBC* |  |  |  |  |  |  | 33.3 |  |  |  | 6.7 | 33 | 93 |  |  |  |  |  |
|  |  | *CYCD2 + HIC1 + PAX5 + RASSF1A + RB1 + SRBC* |  |  |  |  |  |  |  |  |  |  |  | 83.7  (70.5-96.9) | 67.9  (51.2-84.6) |  |  |  |  |  |
| Chang 2010 [12] | P | *ITGA4* | stool | 2007 | 30 | 67.1±7.5 | I-II 47%,  III-IV 53% | 83% distal,  17% proximal | 36.7 | NC | 31 | 58.8±10.2 |  | 36.7 | 100 |  |  |  | QIAamp DNA Stool mini kit | MSP |
|  |  | *p16* |  |  |  |  |  |  | 40 |  |  |  |  | 40 | 96.8 |  |  |  |  |  |
|  |  | *SFRP2* |  |  |  |  |  |  | 60 |  |  |  |  | 60 | 100 |  |  |  |  |  |
|  |  | *ITGA4 + SFRP2 + p16* |  |  |  |  |  |  | 70 |  |  |  |  | 70 | 96.8 |  |  |  |  |  |
| Chen 2017 [13] | P | *SEPT9* | blood | 2012-2013 | 51 |  | 0-II 45%,  III-IV 55% | 92% distal,  8% proximal |  | NC | 9 | 65.8±12.4  (total population) |  | 47 | 89 |  | 96 | 22 | Abbott mSample Preparation System DNA reagent kits | Abbott real- time PCR |
| Chen 2005 [14] | P | *VIM* | stool |  | 94 | 66±12.4 | I-II 64%,  III-IV 36% | 68% distal,  32% proximal |  | H | 198 | 65.5±7.3 |  | 46  (36-56) | 90  (85-94) |  |  |  |  | MSP |
| Church 2014 [15] | P | *SEPT9* | plasma | 2008-2010 | 53 | 67 | I-II 68%,  III-IV 32% |  | 48.2  (32.4-63.6) | H | 938 | 61 | 8.5  (6.9-10.3) | 48.2  (32.4-63.6) | 91.5  (89.7-93.1) |  | 5.2  (3.5-7.5) | 99.5  (99.2-99.6) | Epi proColon | Epi proColon |
|  |  | *SEPT9* |  |  | 51 |  |  |  |  |  |  |  |  | 63.9  (47.5-79.2) | 88.4  (86.2-90.4) |  | 4.82  (3.53-6.73) | 99.63  (99.38-99.79) |  |  |
| deVos 2009 [16] | P | *SEPT9* | plasma |  | 97 | 62.5  (37-87) | I 23%,  II 39%,  III 35%,  IV 3% |  |  |  | 172 | 60 (40-87) |  | 75  (65-83) | 87  (81-91) |  |  |  | Chemagic viral DNA/RNA kit | RT-PCR |
|  |  | *SEPT9* |  |  | 90 | 65  (41-86) | I 21%,  II 44.5%,  III 30%,  IV 4.5% |  |  |  | 155 | 54 (40-90) |  | 72 | 86 |  |  |  |  |  |
| Ebert 2006 [17] |  | *ALX4* | serum |  | 30 | 63.5  (29-81) |  |  |  |  | 30 | 54  (25-78) |  | 83.3 | 70 | 83.9  (72.1-92.1) |  |  | MagnaPure device | MS-APPCR |
|  |  | *ALX4* | serum |  |  |  |  |  |  |  |  |  |  | 90 | 53.3 |  |  |  |  |  |
| Elliott 2013 [18] | P | *APC* | stool |  | 19 | 72±9.36 |  |  |  | NC | 20 | 54.3±12.85 |  |  |  |  |  |  | QIAamp DNA Stool Mini Kit | Q-MSP |
|  |  | *CDH1* |  |  |  |  |  |  |  |  |  |  |  |  |  |  |  |  |  |  |
|  |  | *ESR1* |  |  |  |  |  |  |  |  |  |  |  |  |  |  |  |  |  |  |
|  |  | *MLH1* |  |  |  |  |  |  |  |  |  |  |  |  |  |  |  |  |  |  |
|  |  | *p14arf* |  |  |  |  |  |  |  |  |  |  |  |  |  |  |  |  |  |  |
|  |  | *HPP1 / TMEFF2* |  |  |  |  |  |  |  |  |  |  |  |  |  |  |  |  |  |  |
| Frattini 2008 [19] |  | *p16 INK4a* | plasma |  | 18 | 65  (38-87) | B 36,  C 28,  D 6 | Rectum 33,  Colon 37 | 61.1 |  |  | 60.5  (40-64) |  | 61.1 |  |  |  |  | QIAamp Blood Extraction Kit | fluorescent- methylation specific PCR (F-MSP) |
| Fu 2018 [20] |  | *SEPT9* | plasma | 2015-2016 | 98 |  | 0 3 (3.1%),  I 23 (23.5%),  II 31 (31.6%),  III 31 (31.6%),  IV 8 (8.2%),  UN 2 (2%) | Colon 18.4%;  Rectum 81.6% |  | NED | 253 |  |  | 61.22  (51.33-70.27) | 98.42  (96.01-99.38) | 80.2  (74-86.4) | 93.75  (85-97.54) | 86.76  (82.35-90.2) | Epi proColon  2.0 kit | RT-PCR |
| Gao 2012 [21] | P | *total methylated DNA* | leucocyte s | cohort 1985-88 | 221 | 58  (54-61) |  |  |  | NED | 219 | 58  (54-62) |  |  |  |  |  |  |  | Illumina GoldenGate Methylation Cancer Panel 1 |
| Glockner 2009 [22] | P | *TFPI2* | stool |  | 11 | 60.7 |  |  |  | H | 12 | 54.2 |  | 73  (39-94) | 100  (74-100) |  | 100  (63-100) | 80  (52-96) | Phenol / chloroform | Q-MSP |
|  |  | *TFPI2* | stool |  | 26 | 69.3 |  |  |  | H | 45 | 55 |  | 89  (70-98) | 79  (64-90) |  | 72  (53-86) | 92  (78-98) | QIAamp DNA Stool Mini Kit |  |
|  |  | *TFPI2* | stool |  | 47 | 71.1 |  |  |  | H | 30 | 52.3 |  | 76  (60-88) | 93  (77-99) |  | 94  (80-99) | 73  (56-86) | QIAamp DNA Stool Mini Kit |  |
| Guo 2013 [23] | P | *FBN1* | stool | 2012-2013 | 75 | 58.5±12.5 | I 16%,  II 40%,  III 40%,  UN 4% | 83% distal,  17% proximal |  |  | 30 | 58.4±12.9 |  | 72 | 93.3 |  |  |  | QIAamp DNA Stool Mini Kit | MSP |
| He 2014 [24] |  | *p33ING1b* | stool |  | 61 |  | AB 33,  CD 28 | Rectum 52.5%;  Colon 47.5% |  | NC | 20 | n=30 <=58 years  n=31 >58 years  (total population) |  | 73.77 | 95 |  |  |  | QIAamp DNA Stool Mini Kit | Nested MSP |
| He 2018 [25] | P | *SEPT9* | blood |  | 300 |  | 0 23 (7.7%),  I 42 (14%),  II 99 (33%),  III 124 (41.3%),  IV 12 (0.4%) |  |  | NED | 568 |  |  | 73.7 | 97 |  |  |  | Epi proColon® Plasma Quick Kit | RT-PCR |
| Hellebrekers 2009 [26] | P | *GATA4* | stool |  | 28 | 55 | I 36%,  II 28,5%,  III 28,5%,  IV 7% |  |  |  | 45 | 69 |  | 71  (55-88) | 84  (74-95) | 81  (70-89) |  |  | QIAamp DNA stool midi test kit | Q-MSP |
|  |  | *GATA4* |  |  | 47 | 52 | I 21%,  II 40%,  III 28%,  IV 9%,  UN 2% |  |  |  | 30 | 71 |  | 51  (37-65) | 93  (84-100) |  |  |  |  |  |
| Herbst 2011 [27] |  | *ALX4* | serum |  | 15 | 68  (37-91) |  |  |  |  | 32 |  |  | 46.6 | 66.3 |  |  |  | QIAamp DNA blood mini kit | qMSP |
|  |  | *NEUROG1* |  |  | 15 |  |  |  |  |  | 32 |  |  | 55.5 | 81.3 |  |  |  |  |  |
|  |  | *SEPT9* |  |  | 15 |  |  |  |  |  | 32 |  |  | 46.6 | 81.3 |  |  |  |  |  |
|  |  | *vimentin* |  |  | 15 |  |  |  | I 51.9 %,  II 64.3 % |  | 32 |  |  | 31.1 | 60 |  |  |  |  |  |
|  |  | *NEUROG1* |  |  | 97 | 64  (44-83) | I 27 (27.8%),  II 70 (72.2%) | 39.2% distal  33.0% rectal  27.8% proximal | 94.2 | H | 45 | 63  (44-81) |  | 60.8  (50.4-70.6) | 91.1 | 73.4  (64.9-81.1) |  |  | QIAamp DNA blood mini kit | qMSP |
| Huang 2007 [28] | P | *SFRP2* | stool |  | 52 |  |  |  |  | NC | 24 |  | 4.2 | 94.2 | 95.83 |  |  |  | QIAamp DNA Stool Mini Kit | MSP |
| Huang 2007 [29] | P | *HPP1* | stool |  | 52 | 63.5±11.8 | AB 27 (51.92%),  CD: 25 (48.08%) | 77% distal,  23% proximal |  | NC | 24 | 59.6±8.2 |  | 71.4* | 85.4 |  |  |  | QIAamp DNA Stool Mini Kit | MSP |
|  |  | *MGMT* |  |  |  |  |  |  |  |  |  |  |  | 46* | 93.8 |  |  |  |  |  |
|  |  | *SFRP2* |  |  |  |  |  |  |  |  |  |  |  | 90.5* | 83.3 |  |  |  |  |  |
| Itzkowitz 2008 [30] | P | DY | stool | 2005-2006 | 42 | 67.44±11.21 | I 11 (26.2%),  II 14 (33.3%),  III 14 (33.3%),  IV 3 (7.1%) | 66,7% distal,  33,3% proximal |  | NC | 241 | 56.9±6.33 |  | 60  (44.5-73.0) | 85  (80-89) |  |  |  |  | MSP |
|  |  | hV+DY |  |  |  |  |  |  |  |  |  |  |  | 86  (72.2-93.3) | 73  (67.1-78.2) |  |  |  |  |  |
|  |  | *vimentin* |  |  |  |  |  |  |  |  |  |  |  | 81  (66.7-90.0) | 82  (76.4-86.1) |  |  |  |  |  |
| Itzkowitz 2007 [31] | P | *vimentin* | stool | 2005 | 40 | 65.6±10.3 | I 8 (20%),  II 10 (25%),  III 17 (43%),  IV 5(12%) | 73% distal,  17% proximal |  | NC | 122 | 58.5±7.2 |  | 72.5  (57.2-83.9) | 86.9  (79.8-91.8) |  |  |  |  | MSP |
|  |  | *vimentin* + DIA / DY |  |  |  |  |  |  |  |  |  |  |  | 87.5  (73.9-94.5) | 82  (74.2-87.8) |  |  |  |  |  |
|  |  | *HLTF1* |  |  |  |  |  |  |  |  |  |  |  | 37.5  (24.2-53) | 92.6  (86.6-96.1) |  |  |  |  |  |
| Jin 2015 [32] | P | *SEPT9* | blood | 2013-2014 | 135 | 60.9±12.1 | I 18 (20%),  II 23 (25.6%),  III 44 (48.9%),  IV 5 (5.5%) | Left side 93,  Right side 42 | 74.8 | NC | 91 | 50.1±12.8 |  | 74.8  (67.0-81.6) | 87.4  (83.5-90.6) |  |  |  | Epi proColon 2.0 test | RT-PCR |
| Johnson 2014 [33] | P | *SEPT9* | stool | 2012 | 97 |  | 0 2 (2%),  I 26 (26%),  II 20 (20%),  III 23 (23%),  IV 13 (13%),  UN 17 (17%) |  |  | NED | 193 | 50-84  (total population) |  | 73.3  (63.9-80.9) | 81.5  (75.5-86.3) |  |  |  | Epi proColon test | PCR |
| Kalimutho 2011 [34] | P | *miR-34b/c* | stool |  | 28 | 66  (49-79) | 0 5 (18%),  I 2 (7%),  II 6 (21%),  III 3(11%) | 75% distal,  25%proximal |  | H | 39 | 58 (35-65) | 12.82 | 75 | 87.18 |  |  |  |  | MSP |
| Karam 2018 [35] | P | *p16* | serum / blood |  | 65 | 62.5±11.2 | AB 40 (62.6%),  CD 25 (38.4%) | Colon  26 (41%)  Rectum  39 (58%) |  | H | 70 | 61.6±12.1 |  | 55.38  (43.3-66.8) | 98.5  (92.3-99.7) |  |  |  | QIAamp DNA Blood Mini Kit | MSP |
| Kim 2009 [36] |  | *B4GALT1* | stool |  | 16 |  |  |  |  | NC | 10 |  |  | 56 | 80 | 72±10$ |  |  | Phenol / chloroform / isoamylalcoh ol | MSP |
|  |  | *OSMR* |  |  | 20 |  |  |  |  | NC | 15 |  |  | 45 | 100 | 86±7$ |  |  |  |  |
|  |  | *SFRP4* |  |  |  |  |  |  |  |  |  |  |  | 55 | 100 | 78±7$ |  |  |  |  |
|  |  | *OSMR + SFRP1* |  |  |  |  |  |  |  |  |  |  |  | 60 | 100 | 83±6$ |  |  |  |  |
|  |  | *OSMR* |  |  | 69 |  | I 18 (26%),  II 27 (39%),  III 18 (26%),  IV 6 (9%),  UN 12 (43%) |  |  | H | 81 |  |  | 38 | 95 |  |  |  |  |  |
| Kim 2015 [37] | P | *EYA4* | stool |  | 13 | 58  (42-75) | A 4,  B 4,  C 5 |  | 100 | NED | 19 |  | 5.26 | 100 | 94.74  (75-99) |  |  |  | QIAamp DNA Stool Mini Kit | MSP |
| Kisiel 2018 [38] | CC | *BMP3, VAV3, ZDHHC1* | stool | cohort I: since 2009  cohort II: 1990-1994 |  |  |  |  |  | IBD |  |  |  | 92  (60-100) | 90  (86-93) | 91  (77-100) |  |  | QIAamp DNA Mini Kit | QuARTS  assays |
| Kisiel 2013 [39] | CC | *BMP3* | stool | 2009-2011 | 19 | 60  (45-72) |  | Splenic flexure = 6 |  | IBD | 35 | 60  (45-77) |  | 100 | 91 |  |  |  |  | RT-MSP |
|  |  | *NDRG4 + BMP3* |  |  |  |  |  |  |  |  |  |  |  | 100  (63-100) | 89 |  |  |  |  |  |
| Kitkumthorn 2012 [40] | P | *LINE-1* | blood |  | 36 | 59.47±9.3 |  |  |  | FOC | 144 | 48.67±12.1 |  | 52.78 | 86.81 | 75.5 |  |  | QIAamp DNA Blood Mini Kit | PCR |
| Kriegshauser 2017 [41] | P | *SFRP2* | stool |  | 18 |  | I 2 (11%),  II 16 (89%) | 83% distal,  17% proximal |  | NC | 22 | 57.4  (18-90)  (total population) |  | 61.1 | 86.3 |  |  |  |  | MSRH |
|  |  | *SFRP2* |  |  |  |  |  |  |  |  |  |  |  | 77.7 | 77.3 |  |  |  |  | MethyLight |
| Leclerc 2017 [42] | P | *PDK4* | blood |  | 40 | 59 |  |  |  |  | 40 | 58 |  |  |  |  |  |  | Gentra Puregene Blood Kit |  |
|  |  | *PDK4* |  |  | 18 | 60 |  |  |  |  | 29 | 60 |  |  |  |  |  |  |  |  |
| Lee 2009 [43] | R | *APC +*  *MGM T + RASSF2A + Wif1* | plasma | 2004-2007 | 243 | 61±11 | I 44 (18%),  II 199 (82%) | 77% distal,  23% proximal |  | H | 276 |  |  | 86.5  (81.7-90.8) | 92.1  (88.2-95.0) | 92.7 | 90.6  (86.0-94.1%) | 88.8  (84.4-92.2%) | QIAamp DNA Blood Mini Kit | MSP |
|  |  | *Wif-1* |  |  |  |  |  |  |  |  |  |  |  | 36.7  (30.6-43.7) | 90.6  (86.4-93.1) | 64.1 | 77.5  (68.6-84.7%) | 62.1  (56.9-66.1%) |  |  |
| Lenhard 2005 [44] |  | *HIC1* | stool |  | 26 |  | I 1 (4%),  II 5 (19%),  III 7 (27%),  IV 13 (50%) | 58% distal,  42% proximal |  | NC | 32 |  |  | 42  (23-63) | 100 |  |  |  | QIAamp DNA Stool Mini Kit | MSP |
| Leung 2004 [45] | P | *APC* | stool |  | 20 | 69  (45-90) | B 7,  C 8,  D 4 | 55% distal,  45% proximal | 20 | NC | 20 | age-matched |  |  |  |  |  |  | QIAamp DNA Stool Mini Kit | MSP |
|  |  | *ATM* |  |  |  |  |  |  | 25 |  |  |  |  |  |  |  |  |  |  |  |
|  |  | *HLTF* |  |  |  |  |  |  | 25 |  |  |  |  |  |  |  |  |  |  |  |
|  |  | *hMLH1* |  |  |  |  |  |  | 20 |  |  |  |  |  |  |  |  |  |  |  |
|  |  | *MGMT* |  |  |  |  |  |  | 20 |  |  |  |  |  |  |  |  |  |  |  |
|  |  | *ATM + APC + hMLH1 + HLTF + MGMT* |  |  |  |  |  |  |  |  |  |  |  | 70  (46-88) | 100 |  |  |  |  |  |
| Leung 2005 [46] | P | *APC* | serum |  | 49 | 57  (47-80) | I 5 (11%),  II 15 (33%),  III 17 (38%),  IV 8 (18%) | 51% distal,  45% proximal,  4% synchronous |  | NC | 41 | age-matched |  | 6.1 | 100 | 53 |  |  | QIAamp Blood Kit | Methylight |
|  |  | *HLTF* |  |  |  |  |  |  |  |  |  |  |  | 32.7 | 92.7 | 63 |  |  |  |  |
|  |  | *hMLH-1* |  |  |  |  |  |  |  |  |  |  |  | 42.9 | 97.6 | 71 |  |  |  |  |
| Leung 2007 [47] | P | *APC + ATM + hMLH1 + sFRP2 + HLTF + MGMT + GSTP1* | stool |  | 20 |  |  | 40% distal,  60% proximal |  | NC | 30 | 69  (50-70)  (total population) |  | 75  (50.9-91.3) | 90  (73.5-97.9) |  |  |  | QIAamp DNA Stool Mini Kit | MSP |
|  |  | *COX2* |  |  |  |  |  |  |  |  |  |  |  | 50  (27.2-72.8) | 93  (77.2-99.2) |  |  |  |  |  |
| Li 2015 [48] | P | *FBN1* | stool | 2012-2014 | 89 | <50 n=24,  50-60 n=24,  60-70 n=22,  >70 n=19 | A 17,  B 36,  C 36 | Left=19,  Transverse=5,  Right=8,  Rectum=57 |  | H | 30 |  |  | 70.8 | 93.3 |  |  |  | QIAamp DNA Stool Mini Kit | MSP |
|  |  | *SNCA* |  |  |  |  |  |  |  |  |  |  |  | 70 | 100 |  |  |  |  |  |
|  |  | *SCNA + FBN1* |  |  |  |  |  |  |  |  |  |  |  | 84.3 | 93.3 |  |  |  |  |  |
| Liu 2013 [49] | P | *EYA4* | serum | 2003-2005 | 26 | 67.1  (46-83) |  |  |  | H | 26 | 67.1  (46-83) |  | 57.7 | 90 | 76.8  (63.4-90.1) |  |  |  | qPCR |
|  |  | *SEPT9* |  |  |  |  |  |  |  |  |  |  |  | 50 | 90 | 79.3  (66.6-91.9) |  |  |  |  |
|  |  | *TAC1* |  |  |  |  |  |  |  |  |  |  |  | 57.7 | 90 | 78.9  (66.4-91.4) |  |  |  |  |
|  |  | *TAC1 + EYA4* |  |  |  |  |  |  |  |  |  |  |  | 84.6 | 80.8 | 85.1  (74.2-96.1) |  |  |  |  |
|  |  | *TAC1 + SEPT9* |  |  |  |  |  |  |  |  |  |  |  | 73.1 | 92.3 | 82.1  (70.1-94) |  |  |  |  |
| Lofton-Day 2008 [50] | P | *bisDNA* | plasma |  | 133 | 65 |  | 88% distal  12% proximal |  | NC | 179 |  |  | 15 | 95 | 61 |  |  | MagNa Pure LC Total Nucleic Acid Isolation Kit | HM qPCR |
|  |  | *NGFR* |  |  |  |  |  |  |  |  |  |  |  | 33 | 95 | 70 |  |  |  |  |
|  |  | *SEPT9* |  |  |  |  |  |  |  |  |  |  |  | 52 | 95 | 80 |  |  |  |  |
|  |  | *TMEFF2* |  |  |  |  |  |  |  |  |  |  |  | 30 | 95 | 72 |  |  |  |  |
|  |  | *SEPT9 + NGFR* |  |  |  |  |  |  |  |  |  |  |  |  |  | 81 |  |  |  |  |
|  |  | *TMEFF2 + NGFR + SEPT9 + bisDNA* |  |  |  |  |  |  |  |  |  |  |  |  |  | 79 |  |  |  |  |
| Lu 2014 [51] | P | *GATA4* | stool | 2011-2012 | 56 | 60.6±12.19 | I-II 57,  III-IV 43 | 89% distal  11% proximal | 42.9  (30.77-55.86) |  | 40 |  | 5 | 42.9  (30.77-55.86) | 95  (83.5-98.62) |  |  |  | QiaAmp DNA Stool mini-kit | MSP PCR |
|  |  | *GATA5* |  |  |  |  |  |  | 83.9  (72.19-91.31) |  |  |  | 17.5 | 83.9  (72.19-91.31) | 82.5  (68.05-91.25) |  |  |  |  |  |
|  |  | *NDRG4* |  |  |  |  |  |  | 28.6  (18.42-41.48) |  |  |  | 2.5 | 28.6  (18.42-41.48) | 97.5  (87.12-99.56) |  |  |  |  |  |
|  |  | *SFRP2* |  |  |  |  |  |  | 57.1  (44.14-  69.23) |  |  |  | 10 | 57.1  (44.14-69.23) | 90  (76.95-96.04) |  |  |  |  |  |
| Lu 2014 [51] |  | *VIM* |  |  |  |  |  |  | 41.1  (29.17-54.12) |  |  |  | 15 | 41.1  (29.17-54.12) | 85  (70.93-92.94) |  |  |  |  |  |
|  |  | *SFRP2 + GATA4 + GATA5 + NDRG4 + VIM* |  |  |  |  |  |  | 96.4  (87.88-99.02) |  |  |  | 35 | 96.4  (87.88-99.02) | 65  (49.51-77.87) |  |  |  |  |  |
| Lange 2012 [52] | P | *C9orf50* | plasma | 2008-2011 | 75 |  |  |  |  |  | 66 |  |  |  |  | 70  (64-76) |  |  | QIAamp Circulating Nucleic Acid Kit | MethyLight |
|  |  | *THBD-M* |  |  |  |  |  |  |  |  |  |  |  |  |  | 80  (74-87) |  |  |  | MethyLight |
|  |  | *THBD-M + C9orf50* |  |  |  |  |  |  |  |  |  |  |  |  |  | 80  (73-87) |  |  |  | MethyLight |
|  |  | *C9orf50* | serum | 2008-2011 | 72 |  |  |  |  |  | 6 |  |  |  |  | 69  (64-75) |  |  | QIAamp Circulating Nucleic Acid Kit | MethyLight |
|  |  | *THBD-M* |  |  |  |  |  |  |  |  |  |  |  |  |  | 82  (75-89) |  |  |  | MethyLight |
|  |  | *THBD-M + C9orf50* |  |  |  |  |  |  |  |  |  |  |  |  |  | 83  (76-89) |  |  |  | MethyLight |
| Mayor 2009 [53] | P | *EN1* | stool | 1996-1998 | 30 |  | A 7,  B 6,  C 9,  D 5,  ADE 3 | 83% distal  17% proximal | 26.7 |  | 30 |  | 3.3 | 26.7 | 96.7 |  |  |  |  | RT-PCR |
|  |  | *EN1* | serum |  |  |  |  |  | 13.6 |  |  |  | 0 | 13.6 | 100 |  |  |  |  |  |
| Melotte 2009 [54] | P | *NDRG4* | stool |  | 28 | 69.3±9 | I 35.7%,  II 28.6%,  III 28.6%,  IV 7.1% |  |  | H | 45 | 55±11.5 |  | 61  (43-79) | 93  (90-97) | 77  (66-86) |  |  |  | Q-MSP |
|  |  | *NDRG4* |  |  | 47 | 71.1±9 | I 21.3%,  II 40.4%,  III 27.7%,  IV 2.1% |  |  | H | 30 | 52.3±9.8 |  | 53  (39-67) | 100  (86-100) |  |  |  |  | Q-MSP |
| Melotte 2015 [55] | P | *FOXE1* | plasma | 2007 | 154 | <=65 n=44;  >65 n= 110 | I 28%;  II 28%,  III 30%,  IV 14% |  |  | H | 444 | <=65 n=253;  >65 n=191 |  | 46  (38-54) | 93  (91-95) | 70  (69-73) |  |  | QIAamp Circulating Nucleic Acid Test Kit | Q-MSP |
|  |  | *GATA5* |  |  |  |  |  |  |  |  |  |  |  | 18  (12-24) | 99  (98-100) | 59  (55-63) |  |  |  |  |
|  |  | *NDRG4* |  |  |  |  |  |  |  |  |  |  |  | 27  (20-34) | 95  (93-97) | 61  (57-65) |  |  |  |  |
|  |  | *SYNE1* |  |  |  |  |  |  |  |  |  |  |  | 47  (39-55) | 96  (94-98) | 72  (68-75) |  |  |  |  |
|  |  | *FOXE1 + SYNE1* |  |  |  |  |  |  |  |  |  |  |  | 56  (48-64) | 90  (87-93) |  |  |  |  |  |
|  |  | *FOXE1 + SYNE1* | plasma | 2007 | 66 | <=65 n=24;  >65 n=42 | I 41%,  II 23%,  III 30%,  IV 6% |  |  | H | 240 | <=65 n=105;  >6 5 n=93 |  | 58  (46-70) | 91  (80-100) |  |  |  | QIAamp Circulating Nucleic Acid Test Kit | Q-MSP |
| Melson 2013 [56] | R | *CYCD2* | plasma |  | 30 | 68.3  (49-85) | I 37%,  II 63%,  III 0%,  IV 0% | 73% distal,  27% proximal |  | PE | 30 | 61.2  (40-80) |  | 70 | 73 | 83 |  |  | DNAzol BD and proteinase K | MSRE-PCR |
|  |  | *HIC* |  |  |  |  |  |  |  |  |  |  |  | 77 | 83 | 86 |  |  |  |  |
|  |  | *VHL* |  |  |  |  |  |  |  |  |  |  |  | 76 | 67 | 70 |  |  |  |  |
|  |  | *CYCD2, HIC + VHL* |  |  |  |  |  |  |  |  |  |  |  | 83 | 93 | 94 |  |  |  |  |
|  |  | *CYCD2, HIC + VHL* |  |  |  | 55.9  (45-72) | II 100% | 50% distal,  50% proximal |  | PE |  | 52.6  (50-61) |  | 70 | 90 | 90 |  |  | DNAzol BD and proteinase K | MSRE-PCR |
| Miotto 2004 [57] | P | *CDH4* | plasma |  | 46 |  |  |  | 70 | H | 17 |  | 0 | 70 | 100 |  |  |  | Wizard genomic DNA purification kit | semi-nested PCR |
| Mitchell 2016 [58] | P | *FGF5* | white blood cells | 2006-2011 | 20 | 61  (32-86) | I 5%,  II 38%,  III 31%,  IV 11%,  UN 15% | 65% distal,  35% proximal | 85 | ABD | 40 | 56  (41-83) | 17.5 |  |  |  |  |  | QIAamp circulating nucleic acid kit | MSP |
|  |  | *IRF4* |  |  | 22 |  |  |  | 59.1 |  | 24 |  | 15.9 |  |  |  |  |  |  |  |
|  |  | *PDX1* |  |  | 20 |  |  |  | 45 |  | 20 |  | 30 |  |  |  |  |  |  |  |
|  |  | *SOX21* |  |  | 20 |  |  |  | 85 |  | 20 |  | 50 |  |  |  |  |  |  |  |
|  |  | *GRASP* |  |  | 44 |  |  |  | 54.5 |  | 44 |  | 6.8 |  |  |  |  |  |  |  |
|  |  | *SDC2* |  |  | 44 |  |  |  | 59.1 |  | 44 |  | 15.9 |  |  |  |  |  |  |  |
|  |  | *BCAT1* |  |  | 74 |  |  |  | 64.9 |  | 144 |  | 3.5 |  |  |  |  |  |  |  |
|  |  | *IKZF1* |  |  | 74 |  |  |  | 67.6 |  | 144 |  | 4.9 |  |  |  |  |  |  |  |
|  |  | *SEPT9* |  | 2006-2011 | 44 |  |  |  | 59.1 |  | 44 |  | 4.5 |  |  |  |  |  | QIAamp circulating nucleic acid kit | HeavyMethyl assay |
| Muller 2004 [59] | P | *CALCA* | stool | 2003-2004 | 10 | 67.5  (46-85) |  | 80% distal,  20% proximal | 40 | ABD | 13 | 46  (18-69) | 0 |  |  |  |  |  | QIAamp DNA Stool Kit | MethyLight, real-time PCR |
|  |  | *IGFBP2* |  |  |  |  |  |  | 20 |  |  |  | 0 |  |  |  |  |  |  |  |
|  |  | *PGR* |  |  |  |  |  |  | 80 |  |  |  | 31 |  |  |  |  |  |  |  |
|  |  | *SFRP2* |  |  |  |  |  |  | 90 |  |  |  | 23 | 90  (56-100) | 77  (46-95) |  |  |  |  |  |
|  |  | *SFRP5* |  |  |  |  |  |  | 89 |  |  |  | 46 |  |  |  |  |  |  |  |
|  |  | *CALCA* |  | 2003-2004 | 13 | 63  (34-90) |  | 85% distal,  15% proximal | 31 | ABD | 13 | 47 | 0 |  |  |  |  |  | QIAamp DNA Stool Kit | MethyLight, real-time PCR |
|  |  | *IGFBP2* |  |  |  |  |  |  | 8 |  |  |  | 8 |  |  |  |  |  |  |  |
| Muller 2004 [59] |  | *PGR* |  |  |  |  |  |  | 77 |  |  |  | 31 |  |  |  |  |  |  |  |
|  |  | *SFRP2* |  |  |  |  |  |  | 77 |  |  |  | 23 | 77  (46-95) | 77  (46-95) |  |  |  |  |  |
|  |  | *SFRP5* |  |  |  |  |  |  | 77 |  |  |  | 23 |  |  |  |  |  |  |  |
| Nagai 2017 [60] | P | *LINE-1* | plasma | 2012-2014 | 114 | 63±12.5 | I-II 50%,  III-IV 50% | 70,2% distal,  29,8% proximal |  | NED | 53 | 50.6±19.3 |  | 65.8 | 90 | 81 |  |  | Phenol / chloroform / isoamyl alcohol | AQAMA |
| Nagasaka 2009 [61] | P | *RASSF2 loc1* | stool | 2004-2007 | 84 | 65.2±11.3 | I-II 48%,  III-IV 52% | 66,7% distal,  33,3% proximal | 27.4 | NED | 113 | 66.1±12.5 | 2.7 | 27.4 | 97.3 |  |  |  | QIAmp DNA Mini Kit | High- sensitivity assay for bisulfite DNA (Hi-SA) |
|  |  | *RASSF2 loc2* |  |  |  |  |  |  | 35.7 |  |  |  | 2.7 | 35.7 | 97.3 |  |  |  |  |  |
|  |  | *SFRP2 loc1* |  |  |  |  |  |  | 57.1 |  |  |  | 3.5 | 57.1 | 96.5 |  |  |  |  |  |
|  |  | *SFRP2 loc2* |  |  |  |  |  |  | 38.1 |  |  |  | 4.4 | 38.1 | 95.6 |  |  |  |  |  |
|  |  | combination |  |  |  |  |  |  | 75 |  |  |  | 10.6 | 75 | 89.4 |  |  |  |  |  |
| Grutzmann 2008 [62] | P | *SEPT9* | blood |  | 252 | 61 | I 25%,  II 33%,  III 23%,  IV 11%,  NA 8% | 70% distal,  30% proximal | 48  (41-54) |  | 102 | 59 | 7  (3-14) | 48  (41-54) | 93  (86-97) |  |  |  | Total Nucleic Acid Large Volume DNA extraction kit | PCR |
|  |  | *SEPT9* |  |  | 126 | 67 | I 17%,  II 29%,  III 43%,  IV 9%,  NA 2% | 77% distal,  23% proximal | 58  (49-67) |  | 183 | 56 | 10  (6-15) | 58  (49-67) | 90  (85-94) |  |  |  |  |  |
|  |  | *SEPT9* |  |  |  |  |  |  | 72  (63-80) |  |  |  | 10 (6-16) | 72  (63-80) | 90  (84-94) |  |  |  |  |  |
| Nan 2013 [63] | CC | Genomic DNA methylation | leucocytes | 1989-1990 | 358 | 59 |  |  |  | NED | 661 | 59 |  |  |  |  |  |  |  | ABI PRISM 7900HT Sequence Detction System |
| Nishio 2010 [64] | P | *RUNX3* | serum |  | 344 | 63.9±12.8 | I 19.2%,  II 31.4%,  III 36%,  IV 13.4% | 67% distal,  33% proximal | 29 | H | 56 | 63.9  (38-83) | 0 |  |  |  |  |  | Dneasy Blood & Tissue Kit | RTQ-MSP (2step MSP) |
| Niu 2017 [65] | P | *SDC2* | stool | 2014-2015 | 196 | 61  (43-79) | I-II 44.4%,  III-IV 55.6% | 78,1% distal,  21,9% proximal | 81.1 | H | 179 | 56  (43-77) | 6.7 | 81.1 | 93.3 | 92  (89-95) |  |  | QIAamp DNA Mini Kit | Q-MSP |
|  |  | *SDC2* | stool |  | 19 |  | I-II 44.4%,  III-IV 55.6% |  | 84.2 | H | 20 |  | 5 | 84.2 | 95 |  |  |  |  |  |
| Nunes 2018 [66] | P | *SEPT9* | blood |  | 72 | 63  (29–93) | 0 3 (4%),  I-II 34 (47%),  III-IV 35 (49%) | 32% distal,  68% proximal |  | FA | 103 | 52  (45–65) |  | 11.1 | 98.9 |  | 100 | 61.68 | QIAamp MinElute ccfDNA | multiplex qMSP |
|  |  | *APC* |  |  |  |  |  |  |  |  |  |  |  | 20.83 | 94.17 |  | 71.43 | 62.99 |  |  |
|  |  | *FOXA1* |  |  |  |  |  |  |  |  |  |  |  | 50 | 88.35 |  | 75 | 71.65 |  |  |
|  |  | *RARβ2* |  |  |  |  |  |  |  |  |  |  |  | 16.67 | 95.15 |  | 70.59 | 62.03 |  |  |
|  |  | *RASSF1A* |  |  |  |  |  |  |  |  |  |  |  | 13.89 | 99.03 |  | 90.91 | 62.2 |  |  |
|  |  | *SCGB3A1* |  |  |  |  |  |  |  |  |  |  |  | 26.39 | 90.29 |  | 65.52 | 63.7 |  |  |
|  |  | *SOX17* |  |  |  |  |  |  |  |  |  |  |  | 23.61 | 90.29 |  | 62.96 | 62.84 |  |  |
|  |  | PanCancer panel (*APC, FOXA1, RASSF1A*) |  |  | 37 |  |  |  |  | FA | 103 |  |  | 78.4 | 69.9 |  | 48.3 | 90 |  |  |
| Oh 2013 [67] | R | *SDC2* | serum |  | 131 | 58.4  (33-84) | I 20%,  II 44%,  III 27%,  IV 9% |  |  | NED | 125 | 51  (40-61) |  | 87  (80.0-92.3) | 95.2  (89.8-98.2) | 93  (89-96) |  |  | Dynabeads silane viral NA kit | Q-MSP |
| Oh 2017 [68] | P | *SDC2* | stool |  | 50 | 61.9  (41-84) | I 24%,  II 34%,  III 20%,  IV 22% |  | 90 | H | 22 | 58.8  (36-77) | 9.1 | 90  (78.2-96.6) | 90.9  (70.8-98.6) | 93  (85-98) |  |  | Phenol / chloroform / isoanylalcoho l; QIAamp DNA Stool Mini Kit | LTE-QMSP |
| Orntoft 2015 [69] | R | *SEPT9* | plasma | 2010-2012 | 128 | <=65 58% | I 27,3%,  II 27,3%,  III 23,4%,  IV 22% | 67% distal,  33% proximal | 73  (64-80) | NC | 150 | <=65 65% | 18  (12-25) | 73  (64-80) | 82  (75-88) |  |  |  | Epi proColon Plasma Quick kit | Epi proColon Sensitive PCR kit |
| Pack 2013 [70] | P | *APC* | plasma |  | 60 | 63.4±10.1 | I 23,3%,  II 23,3%,  III 46,7%,  IV 6,7% | 54% distal,  46% proximal |  | AP, H | 60 | 61.4±9.0 |  | 57 | 86 |  | 71 |  |  | MSP–SSCP (single- strand conformation polymorphism) |
|  |  | *DAPK1* |  |  |  |  |  |  |  |  |  |  |  | 50 | 74 |  | 54 |  |  |  |
|  |  | *E-cad (CDH1)* |  |  |  |  |  |  |  |  |  |  |  | 60 | 84 |  | 69 |  |  |  |
|  |  | *FHIT* |  |  |  |  |  |  |  |  |  |  |  | 50 | 84 |  | 65 |  |  |  |
|  |  | *SMAD4* |  |  |  |  |  |  |  |  |  |  |  | 52 | 64 |  | 53 |  |  |  |
| Park 2017 [71] | P | *BMP3* | stool | 2012-2014 | 35 | 60.6±13.04 | I-II 49%,  III-IV 51% | 66% distal,  34% proximal | 40 | NC | 40 | 55.7±14.64 | 15 | 40  (23-57) | 85  (73.4-96.5) |  |  |  | QIAamp DNA stool mini kit | qMSP |
|  |  | *NDRG4* |  |  |  |  |  |  | 68.8 |  |  |  | 20 | 68.8  (52-85) | 80  (67.0-92.9) |  |  |  |  |  |
| Park 2017 [71] |  | *SFRP2* |  |  |  |  |  |  | 60 |  |  |  | 12.5 | 60  (43-77) | 87.5  (76.8-98.2) |  |  |  |  |  |
|  |  | *TFPI2* |  |  |  |  |  |  | 31.4 |  |  |  | 10 | 31.4  (15-48) | 90  (80.3-99.7) |  |  |  |  |  |
|  |  | *SFRP2 + TFPI2 + NDRG4 + BMP3* |  |  |  |  |  |  |  |  |  |  |  | 94.3  (86.2-100) | 55  (38.9-71.1) |  |  |  |  |  |
| Pedersen 2015 [72] | CC | *BCAT1* | plasma |  | 74 | 61  (32-86) | I 5%,  II 38%,  III 31%,  IV 11%,  UN 15% | 65% distal,  35% proximal |  | NC | 144 | 56  (41-83) |  | 64.9  (52.9-75.6) | 96.5  (92.1-98.9) |  |  |  | QIAamp Circulating Nucleic Acid Kit | Q-MSP, hydrolysis probe |
|  |  | *IKZF1* |  |  |  |  |  |  |  |  |  |  |  | 67.6  (55.7-78.0) | 95.1  (90.2-98.0) |  |  |  |  |  |
|  |  | *BCAT1 + IKZF1* |  |  |  |  |  |  |  |  |  |  |  | 77  (65.8-86.0) | 92.4  (86.7-96.4) |  |  |  |  |  |
| Pedersen 2014 [73] | R | *CAHM* | plasma |  | 73 | 59±11.7 | I 16%,  II 29%,  III 32%,  IV 16%,  UN 7% |  | 55 |  | 74 | 52±8.6 | 7 | 55 | 93 |  |  |  | QIAamp Circulating Nucleic Acid Kit | Q-MSP |
| Pedersen 2015 [74] | P | *BCAT1* | plasma | 2011-2014 | 129 | 69  (37-90) | I 22%,  II 33%,  III 31%,  IV 12%,  UN 2% | 50% distal,  40% proximal, | 57  (48-66) | NC | 450 | 58  (40-85) | 5  (3-7) | 57  (48-66) | 95  (93-97) |  |  |  | QIASymphony Circulating Nucleic Acid Kit | Q-MSP |
|  |  | *IKZF1* |  |  |  |  |  |  | 48  (39-57) |  |  |  | 1  (0-2) | 48  (39-57) | 99  (98-100) |  |  |  |  |  |
|  |  | *BCAT1 + IKZF1* |  |  |  |  |  |  | 66  (57-74) |  |  |  | 5  (3-8) | 66  (57-74) | 95  (92-97) |  |  |  |  |  |
| Potter 2014 [75] | R | *SEPT9* | plasma |  | 44 | 50-59 9%.  60-69 55%.  >69 36% | I 39%,  II 27%,  III 23%,  IV 11% |  | 68  (53-80) | NC | 444 | 50-59 45%.  60-69 29%.  >69 27% | 21.2  (19-23) | 68  (53-80) | 78.8  (76.7-80.8) |  | 2.5 | 99.7 | Epi proColon Plasma Quick kit | Epi pro- Colon Sensitive PCR kit |
| Rasmussen 2017 [76] | CC | *ALX4* | plasma | 2003-2005 | 193 | 67.5±11.5 |  |  | 28.5  (22.2-35.4) | RA | 102 | 64.7±14.2 | 1  (0-5.3) | 28.5  (22.2-35.4) |  |  |  |  | The easy- MagTM platform | nested PCR |
|  |  | *APC* |  |  |  |  |  |  | 42  (34.9-49.3) |  |  |  | 32.4  (23.4-42.3) | 42  (34.9-49.3) |  |  |  |  |  |  |
|  |  | *BMP3* |  |  |  |  |  |  | 28.5  (22.2-35.4) |  |  |  | 10.8  (5.5-18.5) | 28.5  (22.2-35.4) |  |  |  |  |  |  |
|  |  | *BNC1* |  |  |  |  |  |  | 11.9  (7.7-17.3) |  |  |  | 12.7  (7.0-20.8) | 11.9  (7.7-17.3) |  |  |  |  |  |  |
|  |  | *BRCA1* |  |  |  |  |  |  | 25.4  (19.4-32.1) |  |  |  | 21.6  (14.0-30.8) | 25.4  (19.4-32.1) |  |  |  |  |  |  |
|  |  | *CDKN2A* |  |  |  |  |  |  | 9.3  (5.6-14.3) |  |  |  | 3.9  (1.1-9.7) | 9.3  (5.6-14.3) |  |  |  |  |  |  |
|  |  | *HIC1* |  |  |  |  |  |  | 5.7  (2.9-10.0) |  |  |  | 1  (0-5.3) | 5.7  (2.9-10.0) |  |  |  |  |  |  |
|  |  | *HLTF* |  |  |  |  |  |  | 11.4  (7.3-16.7) |  |  |  | 3.9'  (1.1-9.7) | 11.4  (7.3-16.7) |  |  |  |  |  |  |
|  |  | *MGMT* |  |  |  |  |  |  | 5.7  (2.9-10.0) |  |  |  | 1  (0-5.3) | 5.7  (2.9-10.0) |  |  |  |  |  |  |
|  |  | *MLH1* |  |  |  |  |  |  | 45.1  (37.9-52.4) |  |  |  | 43.1  (33.4-53.3) | 45.1  (37.9-52.4) |  |  |  |  |  |  |
|  |  | *NDRG4* |  |  |  |  |  |  | 9.3  (5.6-14.3) |  |  |  | 0  (0-3.6) | 9.3  (5.6-14.3) |  |  |  |  |  |  |
|  |  | *NEUROG1* |  |  |  |  |  |  | 20.7  (15.2-27.1) |  |  |  | 19.6  (12.4-28.6) | 20.7  (15.2-27.1) |  |  |  |  |  |  |
|  |  | *NPTX2* |  |  |  |  |  |  | 69.9  (62.9-76.3) |  |  |  | 58.8  (48.6-68.5) | 69.9  (62.9-76.3) |  |  |  |  |  |  |
|  |  | *OSMR* |  |  |  |  |  |  | 11.4  (7.3-16.7) |  |  |  | 6.9  (2.8-13.6) | 11.4  (7.3-16.7) |  |  |  |  |  |  |
|  |  | *PHACTR3* |  |  |  |  |  |  | 14.5  (9.9-20.3) |  |  |  | 5.9  (2.2-12.4) | 14.5  (9.9-20.3) |  |  |  |  |  |  |
|  |  | *PPENK* |  |  |  |  |  |  | 10.4  (6.4-15.6) |  |  |  | 3.9  (1.1-9.7) | 10.4  (6.4-15.6) |  |  |  |  |  |  |
|  |  | *RARB* |  |  |  |  |  |  | 25.4  (19.4-32.1) |  |  |  | 69.6  (59.7-78.3) | 25.4  (19.4-32.1) |  |  |  |  |  |  |
|  |  | *RASSF1A* |  |  |  |  |  |  | 11.4  (7.3-16.7) |  |  |  | 15.7  (9.2-24.2) | 11.4  (7.3-16.7) |  |  |  |  |  |  |
|  |  | *SDC2* |  |  |  |  |  |  | 24.4  (18.5-31.0) |  |  |  | 5.9  (2.2-12.4) | 24.4  (18.5-31.0) |  |  |  |  |  |  |
|  |  | *SEPT9* |  |  |  |  |  |  | 24.4  (18.5-31.0) |  |  |  | 4.9  (1.6-11.1) | 24.4  (18.5-31.0) |  |  |  |  |  |  |
|  |  | *SFRP1* |  |  |  |  |  |  | 21.8  (16.2-28.3) |  |  |  | 6.9  (2.8-13.6) | 21.8  (16.2-28.3) |  |  |  |  |  |  |
|  |  | *SFRP2* |  |  |  |  |  |  | 20.2  (14.8-26.6) |  |  |  | 17.6  (10.8-26.4) | 20.2  (14.8-26.6) |  |  |  |  |  |  |
| Rasmussen 2017 [76] |  | *SPG20* |  |  |  |  |  |  | 15.5  (10.7-21.4) |  |  |  | 11.8  (6.2-19.6) | 15.5  (10.7-21.4) |  |  |  |  |  |  |
|  |  | *SST* |  |  |  |  |  |  | 30.1  (23.7-37.1) |  |  |  | 31.4  (22.5-41.4) | 30.1  (23.7-37.1) |  |  |  |  |  |  |
|  |  | *TAC1* |  |  |  |  |  |  | 52.8  (45.6-60.1) |  |  |  | 47.1  (37.1-57.2) | 52.8  (45.6-60.1) |  |  |  |  |  |  |
|  |  | *TFPI2* |  |  |  |  |  |  | 7.3  (4.0-11.9) |  |  |  | 2  (0.2-6.9) | 7.3  (4.0-11.9) |  |  |  |  |  |  |
|  |  | *THBD* |  |  |  |  |  |  | 9.8  (6.0-14.9) |  |  |  | 1  (0-5.3) | 9.8  (6.0-14.9) |  |  |  |  |  |  |
|  |  | *VIM* |  |  |  |  |  |  | 17.6  (12.5-23.7) |  |  |  | 11.8  (6.2-19.6) | 17.6  (12.5-23.7) |  |  |  |  |  |  |
|  |  | *WIF1* |  |  |  |  |  |  | 9.8  (6.0-14.9) |  |  |  | 3.9  (1.1-9.7) | 9.8  (6.0-14.9) |  |  |  |  |  |  |
|  |  | *WNT5A* |  |  |  |  |  |  | 6.2  (3.3-10.6) |  |  |  | 4.9  (1.6-11.1) | 6.2  (3.3-10.6) |  |  |  |  |  |  |
|  |  | *ALX4, BMP3, NPTX2, RARB, SDC2, SEPT9,*  *VIM +* age + sex |  |  |  |  |  |  |  |  |  |  |  | 90.7 | 72.5 | 86 | 6 | 99.8 |  |  |
| Ravegnini 2015 [77] |  | *SEPT9* | blood | start from 2005 | 27 | 67.5±9.6 |  |  | 61.64 | FTP | 26 | 59.6±5.8 | 79.7 | 92.3 | 66.7 | 77  (64-91) |  |  | QIAamp DNA Mini Kit | Q-MSP |
| Rezvani 2017 [78] | CC | *SPG20* | plasma | 2016 | 37 | 55.97±12.63 |  | 22% distal,  65% proximal | 7.7  (median) (4.15-15.28) | NC | 37 | 55.94±11.84 | 0.59  (median) (0.14-1.12) | 81.1 | 96.9 | 98.4 |  |  | QIAamp DNA Blood Mini Kit | MethyLight |
| Roperch 2013 [79] | P | *NPY* | serum |  | 9 | 67.9±12.9 | I-II 6 (19%),  III-IV 26 (81%) |  |  | NC | 30 | 59.1±16.5 |  | 87 | 80 |  | 47 | 97 | ZR Serum DNA kit | QM-MSP |
|  |  | *PENK* |  |  | 23 |  |  |  |  | NC | 131 |  |  | 78 | 90 |  | 61 | 95 |  |  |
|  |  | *WIF1* |  |  | 32 |  |  |  |  | NC | 161 |  |  | 59 | 95 |  | 70 | 92 |  |  |
| Sabbioni 2003 [80] | P | *TPEF* | leukocyte |  | 29 |  |  |  | 86 | H | 16 |  | 0 | 86* | 100* |  |  |  | Phenol/chloroform, Wizard genomic DNA purification kit | MSP |
| Sakamoto 2010 [81] | P | *p16* | serum |  | 51 | 69.6±1.02 | I 39%,  II 29%,  III 12%,  IV 20% | p16 methylated tumors only: 67% distal, 33% proximal | 59 (MSP),  81 (IP-MSP) | H | 10 | 67.8±2.84 | 0 | 59 (MSP)  81 (IP-MSP) | 100 |  |  |  | QIAamp Boold Kit | MSP |
| Salehi 2015 [82] | P | *ALX4* | serum |  | 25 | 67 |  |  | 68 | NC | 25 |  | 12 | 88 | 68 |  |  |  | QIAamp blood mini kit | MSP |
| Salehi 2012 [83] | P | *SFRP1* | stool |  | 25 | 58±12.87 |  | 40% distal  60% proximal | 52 | H | 25 |  | 8 | 52 | 92 |  |  |  | QIAamp DNA Stool Mini Kit | MSP |
| Shirahata 2014 [84] | P | *vimentin* | serum |  | 242 | VIM positive: 67.48;  VIM negative 67.04 | 0 3%,  I 15%,  II 30%,  III 31%,  IV 20%,  UN 1% | 66% distal  34% proximal | 32.6 | H | 25 |  |  | 32.6 |  |  |  |  | Phenol | qMSP |
|  |  | *CEA + CA19-9 + VIM* |  |  |  |  |  |  |  |  |  |  |  | 55.6 |  |  |  |  |  |  |
| Song 2012 [85] | P | *VIM* | urine |  | 20 | 60.45±13.3 | I 20%,  II 35%,  III 20%,  IV 20% |  | 75 (in LMW DNA) | NC | 20 | 73.8 (7.4) | 10 |  |  |  |  |  | Wizard DNA Isolation Kit | MethyLight |
| Song 2018 [86] | P | *SEPT9* | blood |  | 465 |  | I 52 (11.2%),  II 129 (27.7%),  III 165 (35.5%),  IV 25 (5.4%),  UN 94 (20.2%) |  |  | NED | 610 |  |  | I 53.8,  II 80.6,  III 77.4,  IV 84.2 | 97.2 | 81 |  |  | Epi proColon 2.0 assay | Epi proColon  2.0 assay |
| Suehiro 2018 [87] |  | *TWIST1* | serum | 2015-2017 |  | 71  (41–91) | I 14 (77.8%),  II 1 (5.6%),  III 3 (16.7%) | Right 10 (55.6%),  Left 8 (44.4%) |  | NC |  | 55  (33–79) | 25 | 44.4  (21.5-69.3) | 92 | 65.22 |  |  | MagNA Pure Compact Nucleic Acid Isolation Kit I | combined restriction digital PCR (CORD) |
|  |  | *SEPT9* |  |  |  |  |  |  | 18 |  |  |  |  | 44.4 | 92 | 64.67 |  |  |  |  |
| Suehiro 2018 [88] | P | *TWIST1* | stool |  | 83 | 68  (44-92) | I 31.33%,  II 16.87%,  III 40.96%,  IV 7.23% |  |  | NC | 10 | 55  (41-81) | 0 | 33.7  (23.7-45.0) | 100 |  |  |  | QIAamp DNA Stool Mini Kit | Droplet digital PCR |
| Symonds 2016 [89] | P | *BCAT1 & IKZF1 (panel)* | plasma | 2011-2014 | 66 | 67.4  (42.6-85.4) | I 25.78%,  II 37.88%,  III 25.78%,  IV 10.61% | 54% distal  46% proximal | 33.7  (23.7-45.0) | NED | 1315 | 62.6  (41.1-85.4) | 8.2 | 62.1  (49.3-73.8) | 91.8  (90-93) |  |  |  | QIAsymphon y DNA kit | MSP |
| Symonds 2016 [89] |  |  |  |  |  |  |  |  |  |  |  |  |  |  |  |  |  |  |  |  |
| Takane 2014 [90] | P | *EFHD1* | blood |  | 120 | 67.7±11.4 | I 10%,  II 25%,  III 10%,  IV 55% | 66% distal  34%proximal | 62.1  (49.3-73.8) | ABD | 96 | 63±13.6 | 22 | 63 | 78 |  |  |  | QIAamp DNA Mini Kit | MSP |
|  |  | *PPP1R3C* |  |  |  |  |  |  | 62 |  |  |  | 19 | 81 | 81 |  |  |  | QIAamp DNA Mini Kit | MSP |
|  |  | *PPP1R3C, EFHD1* |  |  |  |  |  |  | 81 |  |  |  | 4 | 53 | 96 |  |  |  |  |  |
|  |  | *PPP1R3C or EFHD1* |  |  |  |  |  |  | 53 |  |  |  | 36 | 90 | 64 |  |  |  |  |  |
| Tang 2011 [91] | P | *SFRP2* | stool |  | 169 |  | I-II 82 (58%),  III-IV 60 (42%) | Methylated cases only:  70% distal  30% proximal | 90 | ABD | 63 |  | 46 | 84 | 54 |  |  |  | QIAamp DNA Stool Mini kit | MSP |
|  |  | *SFRP2* | serum |  | 169 |  | I-II 53 (47%),  III-IV 60 (53%) | Methylated cases only:  71% distal  29% proximal | 84 | ABD | 63 |  | 6 | 66.9 | 93.7 |  |  |  | QIAamp blood mini kit |  |
| Tanzer 2010 [92] | P | *ALX4* | plasma |  | 5 | 59  (27-79) | I 80%;  III 20% |  | 66.9 | H | 22 | 42.5  (25-69) | 18 (cohort 2, 2/3 exp positive),  59 (cohort 2, 1/3 exp positive) |  |  |  |  |  | MagNAPure LC Total Nucleic Acids Large Volume Extaction Kit | qRT-PCR |
|  |  | *SEPT9* |  |  | 28 | 67  (63-71) | 0 18%;  I 7.14%;  IV 50%;  UN 25% |  | 40 (cohort 2, 2/3 exp positive),  80 (cohort 2, 1/3 exp positive) | H | 12 | 60.5  (52-72) | 9 (both cohorts combined, 2/3 exp positive);  12 (both cohorts combined, 1/3 positive) |  |  |  |  |  | MagNAPure LC Total Nucleic Acids Large Volume Extaction Kit | heavy methylight |
|  |  | *SEPT9 & ALX4* panel |  |  | 33 | Cohort 1:  67  (63-71);  Cohort 2:  59  (27-79) | Cohort 1  0 5 (18%),  I 2 (7%),  IV 14 (50%),  UN 7 (25%).  Cohort 2  I 4 (80%),  III 1(20%) |  | 73 (both cohort combined, 2/3 exp positive); 82 (both cohorts combined, 1/3 exp positive) | H | 34 | Cohort 1  60.5  (52-72);  Cohort 2  42.5  (25-69) | 18 (cohort 2, 2/3 exp positive),  5 (cohort 2, 3/6 exp positive) |  |  |  |  |  | MagNAPure LC Total Nucleic Acids Large Volume Extaction Kit | qRT-PCR |
| Toth 2012 [93] | P | *SEPT9* | plasma |  | 92 | 67.8±9.8 | I 27%,  II15%,  III39%,  IV 19% | 39% distal  61% proximal, | 95.6 | NED | 92 | 62.6±9.9 | 15.2 | 95.6  (89.2-98.8) | 84.8  (75.8-91.4) |  |  |  | Epi proColon 2.0 plasma quick kit | RT-PCR |
|  |  | *SEPT9* |  |  | 23 |  |  |  | 79.3 |  | 14 |  | 1 | 79.3  (69.6-87.1) | 98.9  (94.1-100) |  |  |  |  |  |
| Toth 2014 [94] | P | *SEPT9* | plasma |  | 34 | 68.3±9.3 | I 18%,  II 32%,  III 32%,  IV15%,  UN 3% |  | 88.2 | NED | 24 | 48 ±14.9 | 8.3 |  |  |  |  |  | Epi proColon 2.0 Plasma Quick Kit | qRT-PCR |
| Wang 2008 [95] | P | *SFRP2* | stool | 2005-2007 | 69 |  | I-II: 43%,  III-IV: 57% | 70% distal  30% proximal | 87 | NC | 30 |  | 6.7 | 87 | 93.3 |  |  |  | QIAamp DNA Stool Mini Kit | MethyLight |
| Wang 2008 [96] | P | *RASSF1A* | serum | 2006-2007 | 45 |  | I 11%,  II 36%,  III 31%,  IV 22% |  | 28.9 | H | 30 |  | 0 |  |  |  |  |  | QIAamp blood mini kit | MSP |
| Warren 2011 [97] | P | *SEPT9* | plasma | 2008-2009 | 50 | 62  (42-85) | I 14%,  II 62%,  III 14%,  IV 10% | 76.6% distal,  23.4% proximal | All stages: 90;  early-stage 87 | NC | 94 | 58  (40-86) | 12 | 90  (77.4-96.3) | 88  (79.6-93.7) |  | 3.61 | 99.94 | nucleic acid extraction kit | RT-PCR |
| Wu 2016 [98] | P | *SEPT9* | plasma |  | 291 |  | 0 1%,  I 13%,  II 23%,  III 28%,  IV 11%,  UN 24% |  | 76.6  (71.3-81.4) | NC | 295 |  | 4.1  (2.1-7) | 76.6  (71.3-81.4) | 95.9 | 88.2 | 83.8 | 91.1 | BioChain plasma processing kit | MSP |
| Wu 2011 [99] | P | *DLC1* | serum | 2008-2011 | 85 |  | I-II: 46%,  III-IV: 54% | 72% distal  28% proximal | 42.4 | ABD | 45 |  | 8.9 |  |  |  |  |  | QIAamp Blood Mini Kit | MSP |
| Wu 2014 [100] | P | *miR-34a* | stool | 2012-2013 | 82 |  | I 13 (16%) |  | 76.8 | H | 40 |  | 5 | 76.8 | 93.6 |  |  |  | QIAamp DNA Stool Mini Kit | MSP |
|  |  | *miR-34b/c* |  |  |  |  |  |  | 93.6 |  |  |  | 0 | 95 | 100 |  |  |  |  |  |
| Xiao 2015 [101] | P | *NDRG4* | blood | 2010-2011 | 84 | 56  (38-73) | I-II 57.1%,  III-IV 42.9% |  |  | H | 16 | 55.6  (40-74) |  | 54.8 | 78.1 |  |  |  | TGuide Blood Genomic DNA Extraction kit | nested MSP |
|  |  | *NDRG4* | stool |  |  |  |  |  |  |  |  |  |  | 76.2 | 89.1 |  |  |  | Bioneer Stool DNA Extaction kit | nested MSP |
|  |  | *NDRG4* | urine |  |  |  |  |  |  |  |  |  |  | 72.6 | 85 |  |  |  |  | nested MSP |
|  |  | *NDRG4* | urine |  | 76 | 54  (39-71) |  |  | 72.4 | FOC | 36 |  |  |  |  |  |  |  |  |  |
| Xiao 2014 [102] | P | *SFRP2* | stool | 2011-2013 | 40 | 57.5  (41-84) | A 7,  B 19,  C 5,  D 9 | 15% distal  85%proximal | 87.5 | NC | 57 | 59  (43-75) | 9 | 87.5 | 91 |  |  |  | QIAamp DNA Stool Mini Kit | MS-HRM |
|  |  | *VIM* |  |  |  |  |  |  | 55 |  |  |  | 7 | 55 | 93 |  |  |  |  |  |
| Xiao 2014 [102] |  | *SFRP2 + VIM* |  |  |  |  |  |  | 92.5 |  |  |  | 8.8 | 92.5 | 91 |  |  |  |  |  |
| Xie 2018 [103] | P | *SEPT9* | plasma | 2016-2018 | 123 | 66.07 | I 5 (4.9%),  II 36 (35.0%),  III 58 (56.3%),  IV 4 (3.8%) | Colon 77 (62.5%),  Rectum 46 (37.5%) |  | FOC | 125 | 66.17 |  | 61.8  (53.0–69.9) | 89.6  (83.0–93.8) | 75.7  (70.1–80.7) |  |  | EZ DNA Methylation Direct™ Kit | MSP |
| Zhang 2013 [104] |  | *SPG20* | stool |  | 96 | 62.2±9.5 | I 18%,  II 32%,  III 45%,  IV: 4% | Methylated cases only:  77% distal  23% proximal | 80.2 | NC | 30 | 62.9±8.9 | 0 | 80.2 | 100 |  |  |  | QIAamp DNA Stool Mini Kit | MSP |
| Zhang 2012 [105] | P | *TFPI2* | stool | 2008-2009 | 60 | 63.4±4.5 | I-II 47%,  III-IV 53% | 68% distal  32% proximal | 68.3 | NC | 30 | 59.2±8.4 | 0 | 68.3 | 100 |  |  |  | QIAamp DNA Stool Mini Kit | MSP |
| Zhang 2017 [106] | P | *CNRIP1* | blood | 2013-2014 | 50 | 69.35  (48-82) | I-II 54%,  III-IV 46% | 56% distal  44%proximal | 85.5 | H | 20 | 49.23  (37-75) | 63.66 |  |  |  |  |  |  | qMSP |
| Zhang 2007 [107] | P | *SFRP1* | stool |  | 19 | 66  (46-84) | I 52%,  II 17%,  III 24%,  IV 7% | 83% distal  17% proximal | 84 | H | 15 | 59  (36-87) | 14 | CRC: 84;  CRC + adenoma  89  (75-100) | 86  (65-99) |  |  |  | QIAamp DNA Stool Mini Kit | MSP |
| Zhang 2015 [108] | P | *GATA5* | plasma | 2012-2013 | 57 | 56.64±8.27 | I-II 58%,  III-IV 42% | Colon 19,  Rectum 38 | 61.4 | NC | 47 | 61.4±12.41 | 21.28 | 61.4  (47.57-74) | 78.72  (64.34-89.3) |  |  |  | QIAamp DNA Blood Mini Kit | MSP |
|  |  | *ITGA4* |  |  |  |  |  |  | 36.84 |  |  |  | 19.15 | 36.84  (24.45-50.66) | 80.85  (66.74-90.85) |  |  |  |  |  |
|  |  | *SFRP2* |  |  |  |  |  |  | 54.39 |  |  |  | 27.66 | 54.39  (40.66-67.64) | 72.34  (57.36-84.38) |  |  |  |  |  |
|  |  | *GATA5, ITGA4* |  |  |  |  |  |  |  |  |  |  |  | 6.52  (1.37-17.9) | 82.46  (70.09-91.25) |  |  |  |  |  |
|  |  | *GATA5, SFRP2* |  |  |  |  |  |  |  |  |  |  |  | 42.86  (29.71-50) | 91.49  (79.62-90) |  |  |  |  |  |
|  |  | *GATA5 or ITGA4* |  |  |  |  |  |  |  |  |  |  |  | 73.68  (60.34-84.46) | 65.96  (50.69-79.14) |  |  |  |  |  |
|  |  | *GATA5 or SFRP2* |  |  |  |  |  |  |  |  |  |  |  | 73.68  (60.34-84.46) | 65.96  (50.69-79.14) |  |  |  |  |  |
|  |  | *GATA5, SFRP2, ITGA4* |  |  |  |  |  |  |  |  |  |  |  | 15.79  (7.48-27.87) | 93.62  (82.46-98.66) |  |  |  |  |  |
|  |  | *GATA5, SFRP2 or ITGA4* |  |  |  |  |  |  |  |  |  |  |  | 80.7  (68.09-89.95) | 55.32  (40.12-69.83) |  |  |  |  |  |
|  |  | *SFRP2, ITGA4* |  |  |  |  |  |  |  |  |  |  |  | 21.05  (11.38-33.89) | 85.11  (71.69-93.80) |  |  |  |  |  |
|  |  | *SFRP2 or ITGA4* |  |  |  |  |  |  |  |  |  |  |  | 70.18  (56.60-81.57) | 61.7  (46.38-75.49) |  |  |  |  |  |
| Zhou 2017 [109] | P | *PCDH18* | plasma |  | 20 |  | I-II 29%,  III-IV 71% |  | 25.17  (0.02-100)^ |  | 20 |  | 1.655  (0-3.712)^ |  |  | 85 |  |  | QIAamp DNA Midi blood kit | Q-MSP |
| Chen 2019 [110] | R | *BMP3* | stool |  | 41 | 55.9  (28-73) |  |  |  |  | 152 | 46.2 (21-74) |  |  |  | 68.2 |  |  | polyvinylpolypyrrolidone / proteinase K (GenMagBio protocol) | Q-MSP |
|  |  | *NDRG4* |  |  |  |  |  |  |  |  |  |  |  |  |  | 80 |  |  |  |  |
|  |  | *SDC2* |  |  |  |  |  |  |  |  |  |  |  |  |  | 80 |  |  |  |  |
|  |  | *SEPT9* |  |  |  |  |  |  |  |  |  |  |  |  |  | 81.5 |  |  |  |  |
|  |  | *SEPT9, NDRG4, SDC2* |  |  |  |  |  |  |  |  |  |  |  | 90 |  | 92.4 |  |  |  |  |
| Chen 2019 [111] | CC | *SDC2* | serum |  | 111 | 61±12  (25-89) | I: 11.7%  II: 44.2%  III: 35.1%  IV: 6.3%  UN: 2.7% | 54.0% distal  42.3% proximal |  | NED | 114 | 33.2±8.3  (19-60) |  | 71.2  (61.8-79.2) | 95.6  (89.6-98.4) | 88.1  (83.5-92.8) |  |  | cfDNA extraction kit (Suzhou VersaBio Technologies Co. Ltd) | Q-MSP |
|  |  | *SEPT9* |  |  |  |  |  |  |  |  |  |  |  | 73  (63.6-80.8) | 95.6  (89.6-98.4) | 85.4  (80-90.7) |  |  |  |  |
|  |  | *SEPT9, SDC2* |  |  |  |  |  |  |  |  |  |  |  | 86.5  (78.4-92) | 92.1  (85.1-96.1) | 92.2  (88.3-96.1) |  |  |  |  |
| Jensen 2019 [112] | R | *C9orf50* | Plasma | 2014 | 113 | 70.8±9.1 | I:14%  II: 60%  III:20%  IV: 6% | 57.5% distal  42.5% proximal | 76.0 | FTP  NC | 87 | 67.2±6.6 |  | 76.0 | 91.0 | 86 |  |  | QIAamp® Circulating Nucleic Acids kit | ddPCR |
|  |  | *KCNQ5* |  |  |  |  |  |  | 83.0 |  |  |  |  | 83.0 | 95.0 | 91 |  |  |  |  |
|  |  | *CLIP4* |  |  |  |  |  |  | 77.0 |  |  |  |  | 77.0 | 99.0 | 88 |  |  |  |  |
|  |  | *C9orf50, KCNQ5, CLIP4* |  |  |  |  |  |  | 85.0 |  |  |  |  | 85.0 | 99.0 |  |  |  |  |  |
| Li 2019 [113] | CC | *SFRP2* | Serum |  | 62 |  | I:21%  II: 45%  III:27%  IV: 5% | 43.0% distal  52.0% proximal  5% UN | 69.4 | NED | 55 |  | 12.7 | 69.4  (56.2-80.1) | 87.3  (74.9-94.3) | 82.1 |  |  | cfDNA extraction kit (VersaBio Technologies Co. Ltd.) | MethylLight |
| Ma 2019 [114] | P | *SEPT9* | Plasma |  | 117 | 67.3±12.8 | I:17%  II: 40%  III:30%  IV: 3%  UN : 9% | 78.6% distal  20.4% proximal  1%synchronous | 73.2  (65.0-81.1) | NC | 70 | 63.1±11.7 | 29.0 | 73.2  (65.0-81.1) | 71.0 |  |  |  | Epi proColon 2.0 Epigenomics AG | Epi proColon 2.0 |
| Pakbaz 2019 [115] | CC | *VIM* | Stool | 2017 | 49 | 61.2 |  |  | 60.0 | NED | 30 | 58.8 | 0 | 60.0 | 100 |  | 100 | 65.2 | QIAamp DNA Stool Mini Kit | MSP |
| Pasha 2019 [116] | CC | *SFRP1* | serum |  | 85 |  | I:11%  II: 46%  III:40%  IV: 3% |  | 77.6 | NED | 40 |  | 0 | 77.7 | 70.0 |  | 84.6 | 59.8 | QIAamp DNA Stool Mini Kit | MSP |
|  |  | *RUNX3* |  |  |  |  |  |  | 60.0 |  |  |  | 0 | 60.0 | 82.5 |  | 87.9 | 49.3 |  |  |
|  |  | *SFRP1 + RUNX3* |  |  |  |  |  |  |  |  |  |  |  | 80.0 | 70.0 |  | 85.0 | 62.2 |  |  |
|  |  | *SFRP1, RUNX3 + CEA* |  |  |  |  |  |  |  |  |  |  |  | 84.7 | 67.5 |  | 84.7 | 67.5 |  |  |
| Picardo 2019 [117] | CC | *B4GALT1* | Plasma | 2008-2016 | 54 | 64  (38-93) |  | 70.4% distal  29.6% proximal | 46.0 |  | 19 |  | 0 | 50.0 | 100 | 75.0  (59.2-90.8) |  |  | phenol chloroform | Q-MSP |
| Salama 2019 [118] | CC | *P14^ARF^* | Blood | 2016-2018 | 60 | 48±11 | I:18%  II: 72%  III:10% | 41.6% distal  58.3% proximal | 60.0 |  | 30 | 45±9 | 13.3 | 60.0 | 86.7 |  | 90 | 52.0 | QIAamp® Spin Columns (QIAGEN Inc. CA 91355, USA) | MSP |
|  |  | *RASSF1A* |  |  |  |  |  |  | 55.0 |  |  |  | 3.3 | 55.0 | 96.7 |  | 97.1 | 51.8 |  |  |
| Sun 2019 [119] | P | *SEPT9* | Plasma |  | 63 |  |  |  | 73.0 | NED | 494 |  | 5.5 | 73.0  (60.1-83.1) | 94.5  (92.0-96.3) | 83.5  (75.8-91.3) | 63.0  (50.9-73.8) | 96.5  (94.3-97.9) | BioChain Science and Technology, Inc. (Beijing) | Epi proColon 2.0 |
| Sun 2019 [120] | CC | *BMP3* | Stool |  | 105 |  | I:15%  II: 31%  III:33%  IV: 12%  UN:8% | 75.2% distal  14.3% proximal  10.5% UN |  | NC | 102 |  |  | 37.1 | 89.8 | 62.0 |  |  | QIAamp Fast DNA Stool mini kit | MethylLight |
|  |  | *SFRP2* |  |  |  |  |  |  |  |  |  |  |  | 38.6 | 90.7 | 78.8 |  |  |  |  |
|  |  | *NDRG4* |  |  |  |  |  |  |  |  |  |  |  | 46.7 | 90.7 | 68.1 |  |  |  |  |
|  |  | *SDC2* |  |  |  |  |  |  |  |  |  |  |  | 68.6 | 91.7 | 80.0 |  |  |  |  |
| Zhao 2019 [121] | CC | *SDC2* | Plasma |  | 117 | 61.8  (28-89) | I:17%  II: 43%  III:32%  IV: 3%  UN :4% | 45.3% distal  52.1% proximal  2.6% UN | 69.2 | NC | 166 | 36.6  (21-69) | 4.2 | 69.2  (59.9-77.3) | 95.8  (91.2-98.1) | 88.6  (84.3-93.0) |  |  | cfDNA extraction kit (Suzhou VersaBio) | Q-MSP |
|  |  | *SEPT9* |  |  |  |  |  |  | 82.1 |  |  |  | 4.2 | 82.1  (73.6-88.3) | 95.8  (91.2-98.1) | 90.0 |  |  |  |  |
|  |  | *SDC2 + SEPT9* |  |  |  |  |  |  | 88.9 |  |  |  | 7.2 | 88.9  (81.4-93.7) | 92.8  (87.4-96.0) | 94.1  (90.9-97.3) |  |  |  |  |
| Bagheri 2020 [122] | CC | *TFPI2* | Blood |  | 50 | 55.8  (24-83) | I:32%  II: 38%  III:18%  IV: 12% |  | 43.9 | NC | 50 | 54.4  (30-80) | 11.6 | 88.0 | 92.0 | 94.0 |  |  | Genomic DNA Isolation kit (GeNetBio) | MethyQESD |
|  |  | *NDRG4* |  |  |  |  |  |  | 38.8 |  |  |  | 12.2 | 86.0 | 92.0 | 95.0 |  |  |  |  |
|  |  | *TFPI2 + NDRG4* |  |  |  |  |  |  |  |  |  |  |  |  |  | 96.1 |  |  |  |  |
| Bhat 2019 [123] | CC | *PARK2* | Blood |  | 200 |  | I-II: 36%  III-IV: 64% |  | 33.0 | H | 200 |  |  | 33.0 |  |  |  |  |  | MSP |
| Bi 2019 [124] | P | *CHST7* | Blood | 2004-2010 | 478 | 60±11.5 | A: 9%  B: 49%  C: 35%  D: 7% |  |  | FOC | 462 | 58±11 |  |  |  |  |  |  | QIAamp DNA Blood Mini kit | MS-HRM |
| Constancio 2019 [125] | CC | *SEPT9* | Plasma | 2015-2019 | 100 | 66  (27-93) | I-II: 39%  III-IV: 61% | 77.0% distal  23.0% proximal |  | A | 136 | 57  (45-84) |  | 8.0  (4.0-15.0) | 100 | 53.3  (45.8-60.8) |  |  | QIAmp MinElute ccfDNA (Qiagen) | Q-MSP |
|  |  | *SOX17* |  |  |  |  |  |  |  |  |  |  |  | 11.0  (6.0-19.0) | 100 | 55.0  (47.5-62.5) |  |  |  |  |
|  |  | *SEPT9 + SOX17* |  |  |  |  |  |  |  |  |  |  |  | 12.0  (6.0-20.0) | 100 |  |  |  |  |  |
| Kerachian 2020 [126] | CC | *CLDN1, INHBA + SLC30A10* | Plasma |  | 22 | 67  (44-82) |  | 41.0% distal  59.0% proximal | 41.0 |  | 20 | 56  (45-84) | 0 | 41.0 | 100 |  |  |  | QIAamp Circulating Nucleic Acid kit | MethylLight |
| Yong-Suk 2019 [127] | CC | *SFRP2* | Serum | 2008-2010 | 68 |  |  |  | 86.8 | H | 60 |  | 0 | 86.8 | 100 |  |  |  |  | MSP |
|  |  | *SFRP2* | Stool |  |  |  |  |  | 89.7 |  |  |  | 0 | 89.7 | 100 |  |  |  | phenol chloroform isoamyl alcohol |  |
| Liu 2020 [128] | P | *COL4A1* | Stool | 2014-2016 | 80 | 60  (46-78) | I-II: 54%  III-IV: 46% | 79.0% distal  21.0% proximal | 88.8 | H | 83 | 58  (45-77) | 12.0 | 88.8 | 88.0 | 96.5  (94.1-98.9) |  |  |  | Q-MSP |
|  |  | *COL4A2* |  |  |  |  |  |  | 92.5 |  |  |  | 8.4 | 92.5 | 91.6 | 96.6  (94.1-99.1) |  |  |  |  |
|  |  | *ITGA4* |  |  |  |  |  |  | 82.5 |  |  |  | 3.6 | 82.5 | 96.4 | 95.3  (92.0-98.5) |  |  |  |  |
|  |  | *TLX2* |  |  |  |  |  |  | 88.8 |  |  |  | 3.6 | 88.8 | 96.4 | 95.6  (92.3-98.9) |  |  |  |  |
|  |  | *COL4A2 + TLX2* |  |  |  |  |  |  | 91.3 |  |  |  | 2.4 | 91.3 | 97.6 | 98.3  (96.7-99.9) |  |  |  |  |
|  |  | *COL4A1* |  |  | 18 | 62  (47-77) | I-II: 33%  III-IV: 67% | 61.0% distal  39.0% proximal | 83.0 |  | 18 | 59  (48-74) | 6.0 | 83.0 | 94.0 | 95.2  (88.2-100) |  |  |  |  |
|  |  | *COL4A2* |  |  |  |  |  |  | 100.0 |  |  |  | 6.0 | 100 | 94.0 | 99.1  (96.6-100) |  |  |  |  |
|  |  | *ITGA4* |  |  |  |  |  |  | 94.0 |  |  |  | 6.0 | 94.0 | 94.0 | 94.0  (84.8-100) |  |  |  |  |
|  |  | *TLX2* |  |  |  |  |  |  | 89.0 |  |  |  | 6.0 | 89.0 | 94.0 | 94.6  (86.7-100) |  |  |  |  |
| Liu 2020 [129] | CC | *SEPT9* | Stool | 2018-2019 | 72 | 60  (35-86) | I:21%  II: 25%  III:32%  IV: 12%  UN : 10% | 45.8% distal  45.8% proximal  8.4% UN |  | NED | 76 | 45  (16-67) |  | 1/3 replicates 97,2; 2/3 replicates 88,9; 3/3 replicates 86,1; 3/3 replicates plus Ct <40 83,3 | 1/3 replicates 48,7; 2/3 replicates 69,7; 3/3 replicates 86,9; 3/3 replicates plus Ct<40 92,1 | 93.5  (89.5-97.5) |  |  | stool DNA extration kit Suzhou Versabio Tech | Q-MSP |
|  |  | *SEPT9* | Plasma |  | 90 | 61  (28-86) | I:20%  II: 30%  III:29%  IV: 11%  UN : 10% | 43.3% distal  45.6% proximal  11.1% UN |  |  | 81 | 42  (21-76) |  | 1/3 replicates: 85,6; 2/3 replicates 64,4; 3/3 replicates 53,3; 3/3 replicates plusCt<40 46,7 | 1/3 replicates 90,1; 2/3 replicates 93,8; 3/3 replicates 96,3; 3/3 replicates plus Ct<40 98,8 | 88.5  (83.2-93.8) |  |  |  |  |
| Luo 2020 [130] | R | *CG10673833* | Blood | 2015-2017 | 801 | 58  (24-85) | I:5%  II: 17%  III:26%  IV: 51%  UN : 1% |  |  | H | 1021 | 47  (19-90) |  |  |  |  |  |  | Elite health cf DNA extraction kit | ddPCR |
|  |  | *CG10673833* |  |  | 29 | (45-75) |  |  | 89.7 | NC | 1386 | (45-75) | 13.2 | 89.7  (72.7-97.8) | 86.8  (84.9-88.4) | 90.0  (88.5-94.2) | 11.8  (10.1-13.8) | 99.8  (99.3-99.9) |  |  |
| Suehiro 2020 [131] | P | *TWIST1* | Stool | 2007-2019 | 205 | 69  (33-92) | I:34%  II: 25%  III:38%  IV: 3% | 66.3% distal  33.7% proximal | 44.4 | NC | 71 | 51  (33-79) | 8.5 | 44.4  (37.4-51.5) | 91.5  (82.5-96.8) |  |  |  | QIAamp DNA Stool Mini kit (Qiagen) | CORD |
|  |  | *TWIST1 +* FIT |  |  |  | 70  (33-93) |  |  |  |  |  |  |  | 95.6  (91.8-98.0) | 80.3  (69.1-88.8) |  |  |  |  |  |
| Vega-Benedetti 2020 [132] | CC | *GRIA4* |  |  | 10 |  | 0: 10%  I:10%  II: 30%  III:30%  IV: 20% | 20.0% distal  80.0% proximal | 40.0(Q-MSP)  90.0(ddPCR) |  |  |  |  |  |  |  |  |  | QIAamp DNA Stool Mini Kit (Qiagen) | MethylLight |
|  |  | *VIPR2* | Stool |  |  |  |  |  | 70.0(Q-MSP)  90.0(ddPCR) |  |  |  |  |  |  |  |  |  |  |  |
| Yang 2020 [133] | CC | NDRG4 | Stool | 2016-2020 | 50 | 60.2±13.6 | I-III: 74%  IV: 26% | 30.0% distal  70.0% proximal |  | NC | 50 | 64.9±11 |  |  |  | 94.9 |  |  | sequence‑specific DNA captures and magnetic beads‑based oligonucleotides | Q-MSP |
|  |  | *SDC2* |  |  |  |  |  |  |  |  |  |  |  |  |  | 99.2 |  |  |  |  |
|  |  | *TFPI2* |  |  |  |  |  |  |  |  |  |  |  |  |  | 96.9 |  |  |  |  |
|  |  | *NDRG4, SDC2, TFPI2, KRAS +* β-actin |  |  |  |  |  |  |  |  |  |  |  | 90.0 | 94.0 | 94.8 |  |  |  |  |
| Zhao 2020 [134] | CC | *SFRP2* | Plasma |  | 122 | 61.8  (30-88) | I:16%  II: 31%  III:36%  IV: 7%  UN : 11% | 51.6% distal  45.1% proximal  3.3% UN |  | NED  NC | 91 | 39.3  (23-69) |  | 63.1  (53.9-71.5) | 90.1  (81.6-95.1) | 78.7  (72.9-84.6) |  |  | cfDNA extraction kit (Suzhou VersaBio) | Q-MSP |
|  |  | *SDC2* |  |  |  |  |  |  |  |  |  |  |  | 56.6  (47.3-65.4) | 95.6  (88.5-98.6) | 76.5  (70.4-82.6) |  |  |  |  |
|  |  | *SFRP2 + SDC2* |  |  |  |  |  |  |  |  |  |  |  | 76.2  (67.5-83.3) | 87.9  (79.0-93.5) | 85.6  (80.6-90.5) |  |  |  |  |
| Zhao 2020 [135] | CC | *SEPT9 (test)* | Stool | 2018-2019 | 55 | 60  (35-86) | 0: 2%  I:20%  II: 27%  III:33%  IV: 7%  UN : 11% | 44.0% distal  49.0% proximal  7% UN |  | NC | 65 | 45.2  (24-69) | 4.6 | 85.5  (72.8-93.1) | 95.4 | 89.2  (83.1-95.3) |  |  | stool DNA extraction kit (Suzhou VersaBio Technologies Co., Ltd.) | Q-MSP |
|  |  | *SDC2*  *(test)* |  |  |  |  |  |  |  |  |  |  | 6.2 | 83.6  (70.7-91.8) | 93.8 | 93.0  (87.7-98.3) |  |  |  |  |
|  |  | *SEPT9 + SDC2*  *(test)* |  |  |  |  |  |  |  |  |  |  | 9.2 | 89.1  (77.1-95.5) | 90.8  (80.3-96.2) | 95.6  (92.4-98.8) |  |  |  |  |
|  |  | *SEPT9*  *(validation)* |  |  | 39 | 59  (27-83) | I:23%  II: 20%  III:41%  IV: 8%  UN: 8% | 44.0% distal  49.0% proximal  7% UN |  |  | 59 | 47.9  (24-83) |  | 82.1  (65.9-91.9) | 96.6  (87.3-99.4) | 94.8  (90.1-99.5) |  |  |  |  |
|  |  | *SDC2*  *(validation)* |  |  |  |  |  |  |  |  |  |  |  | 87.2  (71.8-95.2) | 96.6  (87.3-99.4) | 93.7  (87.5-99.9) |  |  |  |  |
|  |  | *SEPT9 + SDC2*  *(validation)* |  |  |  |  |  |  |  |  |  |  |  | 92.3  (78.0-98.0) | 93.2  (82.7-97.8) | 97.7  (95.2-100) |  |  |  |  |
| Han 2019 [136] | CC | *SDC2* | Stool | 2017-2018 | 245 |  | 0: 1%  I:22%  II: 29%  III:39%  IV: 9% | 76.5% distal  18.0% proximal  5.5% other | 90.0 | NED  NC | 245 |  | 9.8 | 90.2  (85.8-93.6) | 90.2  (85.8-93.6) | 90.2  (87.6-92.8) |  |  | MaXtract High Density tube (Qiagen) and phenol-chloroform-isoamylalcohol | LTE-Q-MSP |

N=number of samples, Se=sensitivity, Sp=specificity, AUC=area under the curve, ROC=receiver operating characteristic curve, PPV=positive predictive value, NPV=negative predictive value, CI=confidence interval, F=forward primer, R=reverse primer, MF=methylated forward primer, MR=methylated reverse primer, UF=unmethylated forward primer, UR=unmethylated reverse primer, M=methylated probe sequence, U=unmethylated probe sequence, IM=internal primer specific for methylated alleles; IU=internal primer specific for unmethylated alleles, PCR=polymerase chain reaction, MSP=methylation specific PCR, qMSP=quantitative MSP, AQAMA=Modified absolute quantitative analysis of methylated alleles assay, qRT-PCR=quantitative real-time PCR, Hi-SA=High-sensitivity assay for bisulfite DNA, MS-APPCR=methylation-specific arbitrarily primed polymerase chain reaction, QuARTS assays=Quantitative Allele-Specific Real-Time Target and Signal Amplification Assays, P=prospective, R=retrospective, CC=case-control, NC=normal colonoscopy, NED=no evidence of disease, IBD=Inflammatory bowel disease without neoplasia; ABD=allowing benign disease; FOC=free of cancer; PE=Polyps excluded; AP=Adenomatous polyp with low-grade dysplasia; RA=Resectable adenomas included; FTP=FOBT test positive; H=healthy; (F)A=(Female) asymptomatic; Se=sensitivity, Sp=specificity

* Sensitivity of detecting cancer and precancerous lesions (including 10 advanced adenomas and 1 unclassfied with dysplasia),

# numbers between brackets in the column indicate SD or range,

$ study provides AUC with SD,

^ study provides the methylation percentage of the corresponding gene in tumor or control samples within a range.
